# Supplementary figures and images for: Taxonomic and chemical assessment of exceptionally abundant rock mine biofilm
Source: PeerJ. 2017 Aug 15;5:e3635. doi: 10.7717/peerj.3635 (PMC5562143; doi:10.7717/peerj.3635)

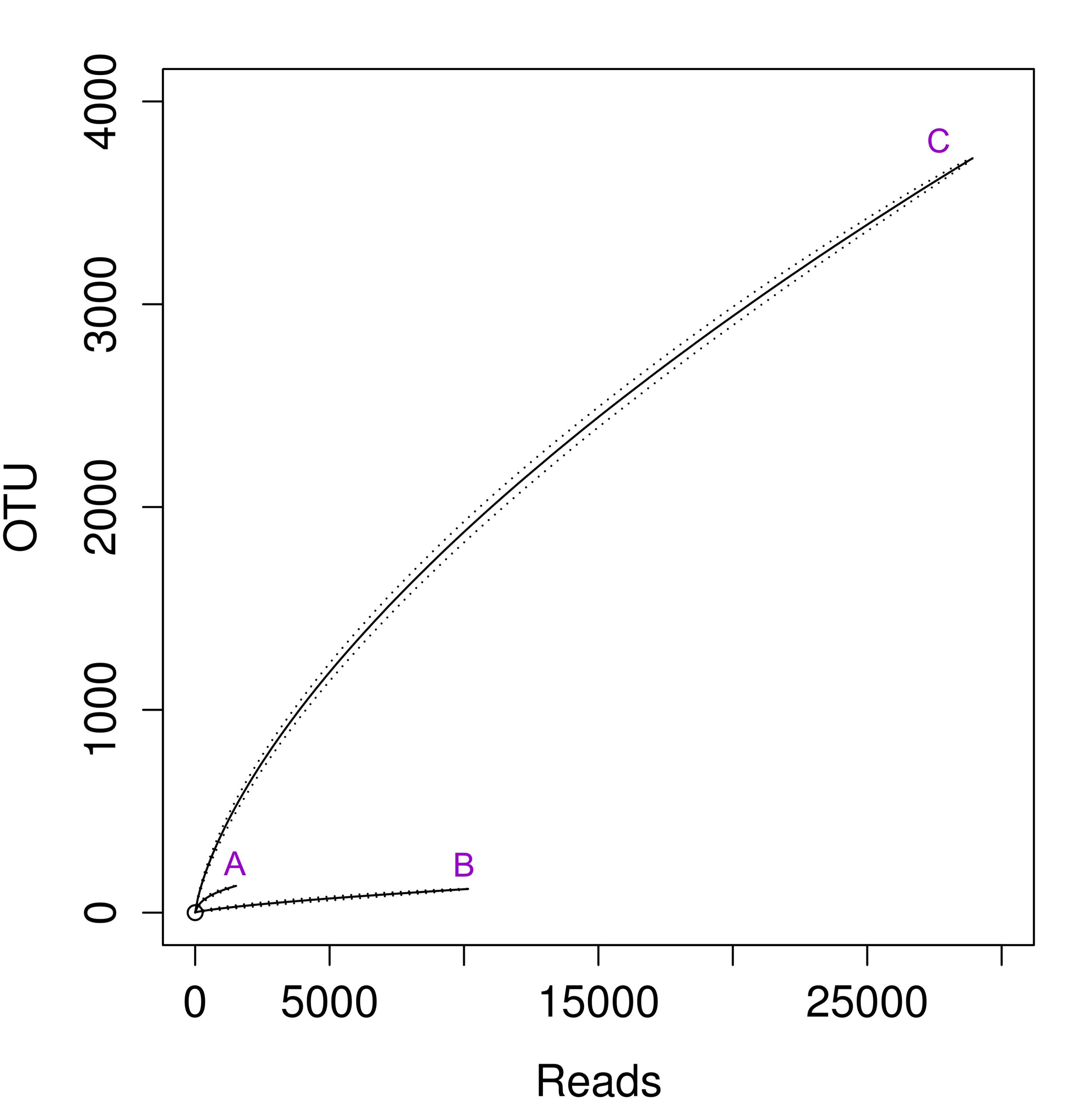

Supplement: Figure S1 — (A) Actinobacteria, (B) Archaea, (C) universal. Curves were calculated at 2% evolutionary distance. [file peerj-05-3635-s001.jpg]

# ENERGY SOURCE

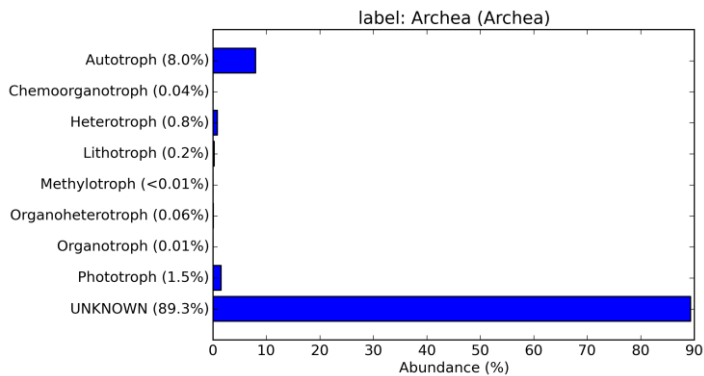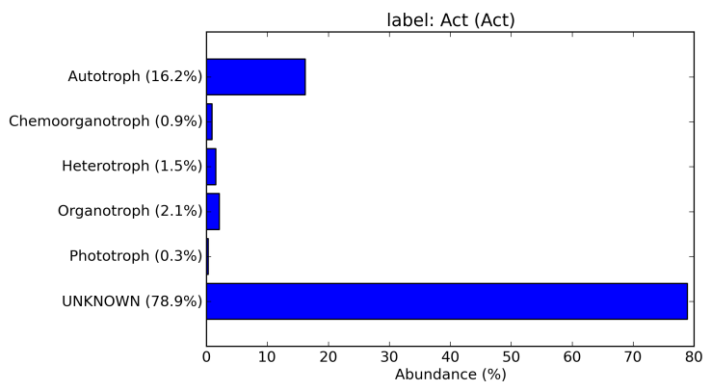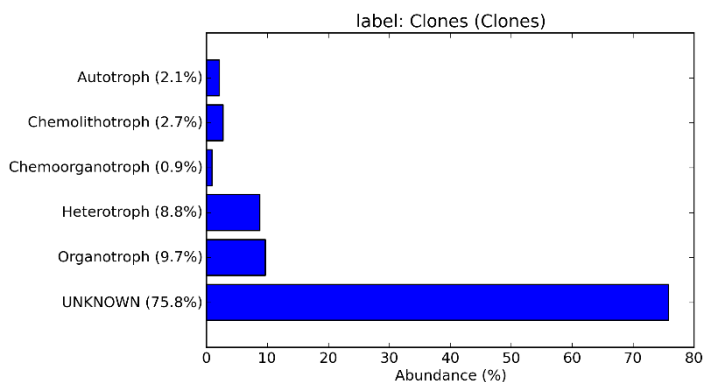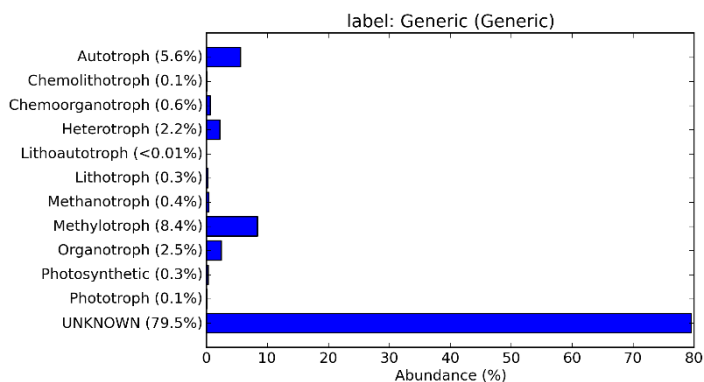

# OXYGEN REQUIREMENT

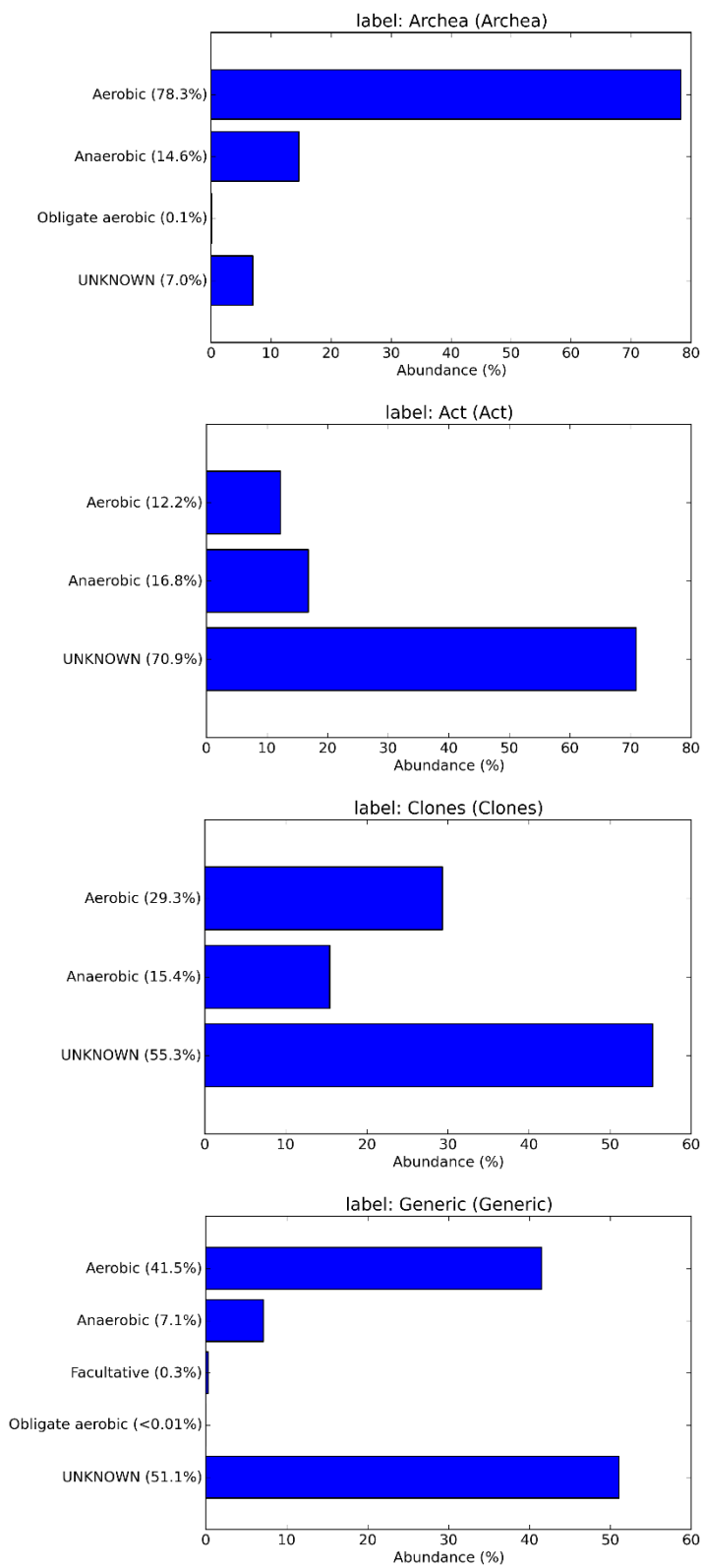

# METABOLISM

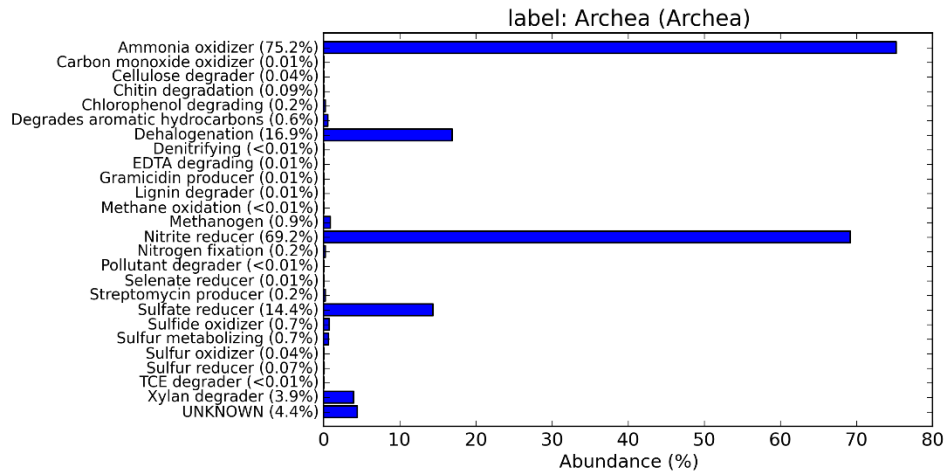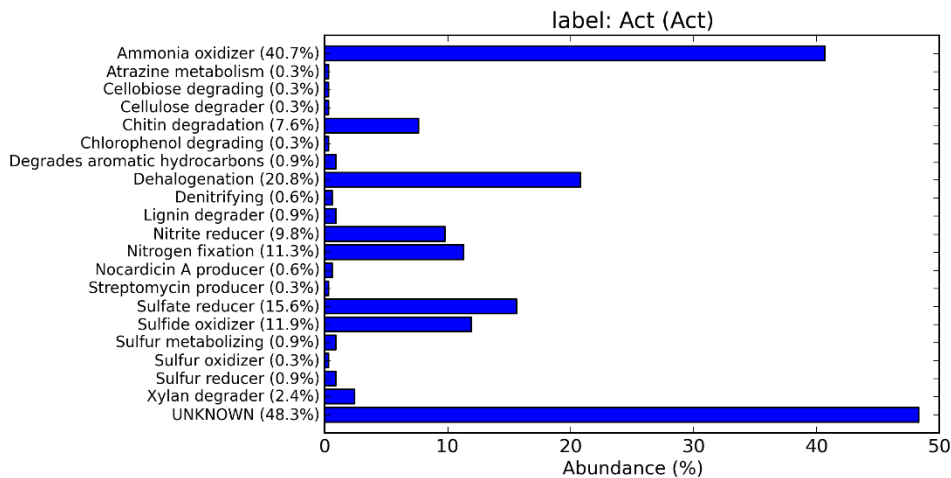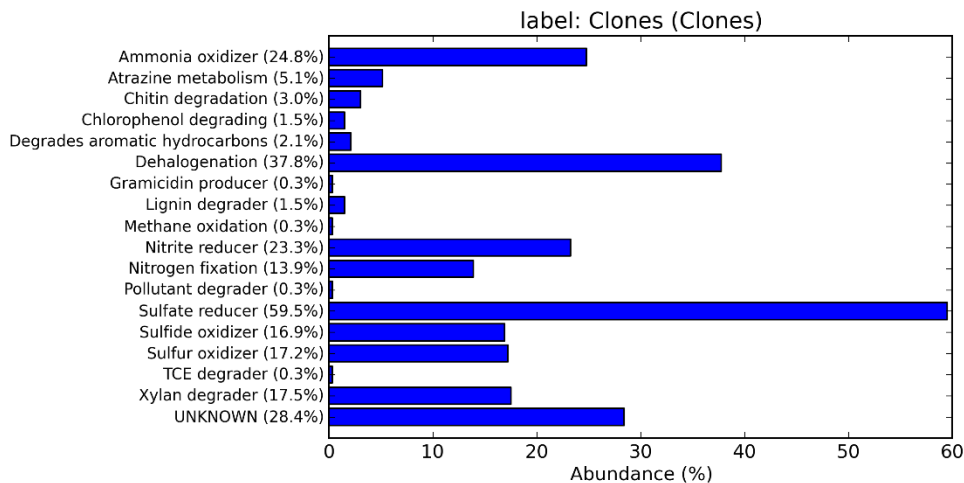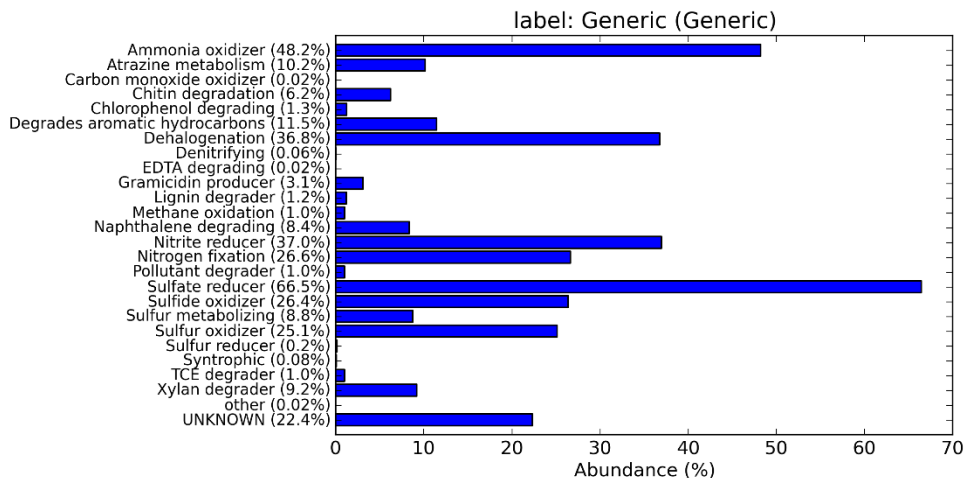

# BIOTIC RELATIONSHIP

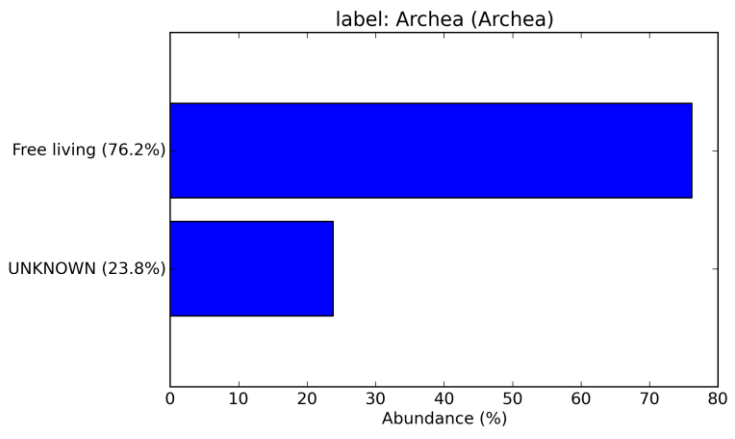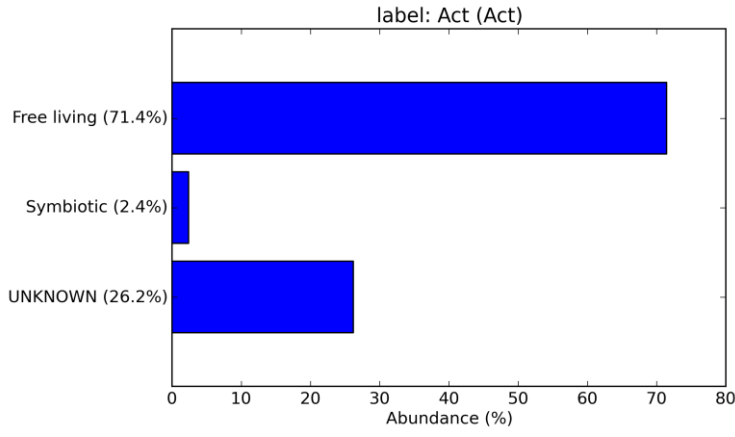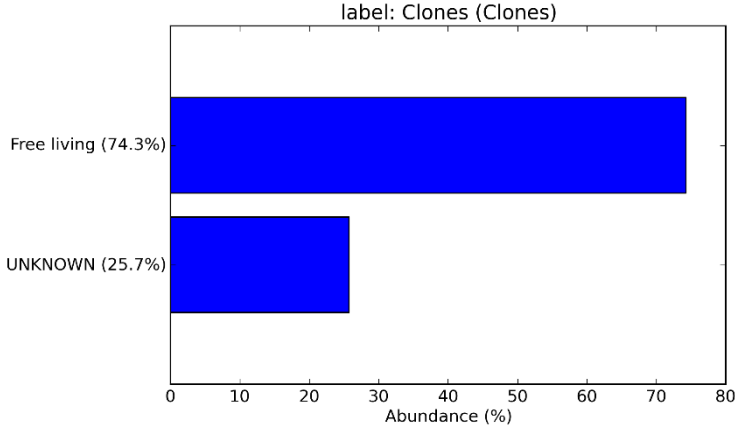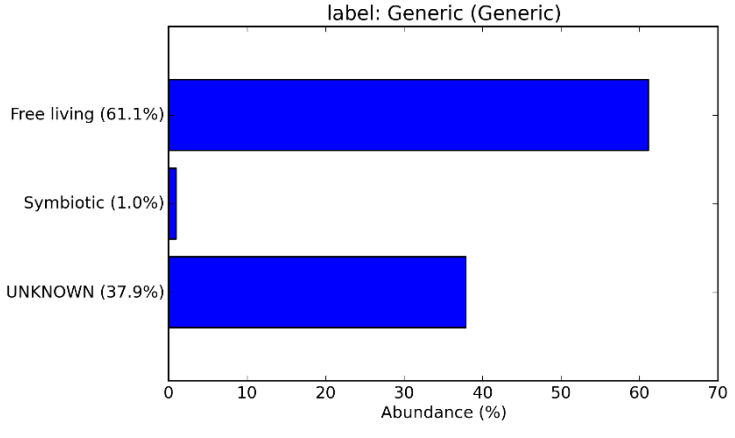

Supplement: Figure S2 — The graphs show: energy sources, oxygen requirements, metabolism and biotic relationships. [file peerj-05-3635-s002.pdf]

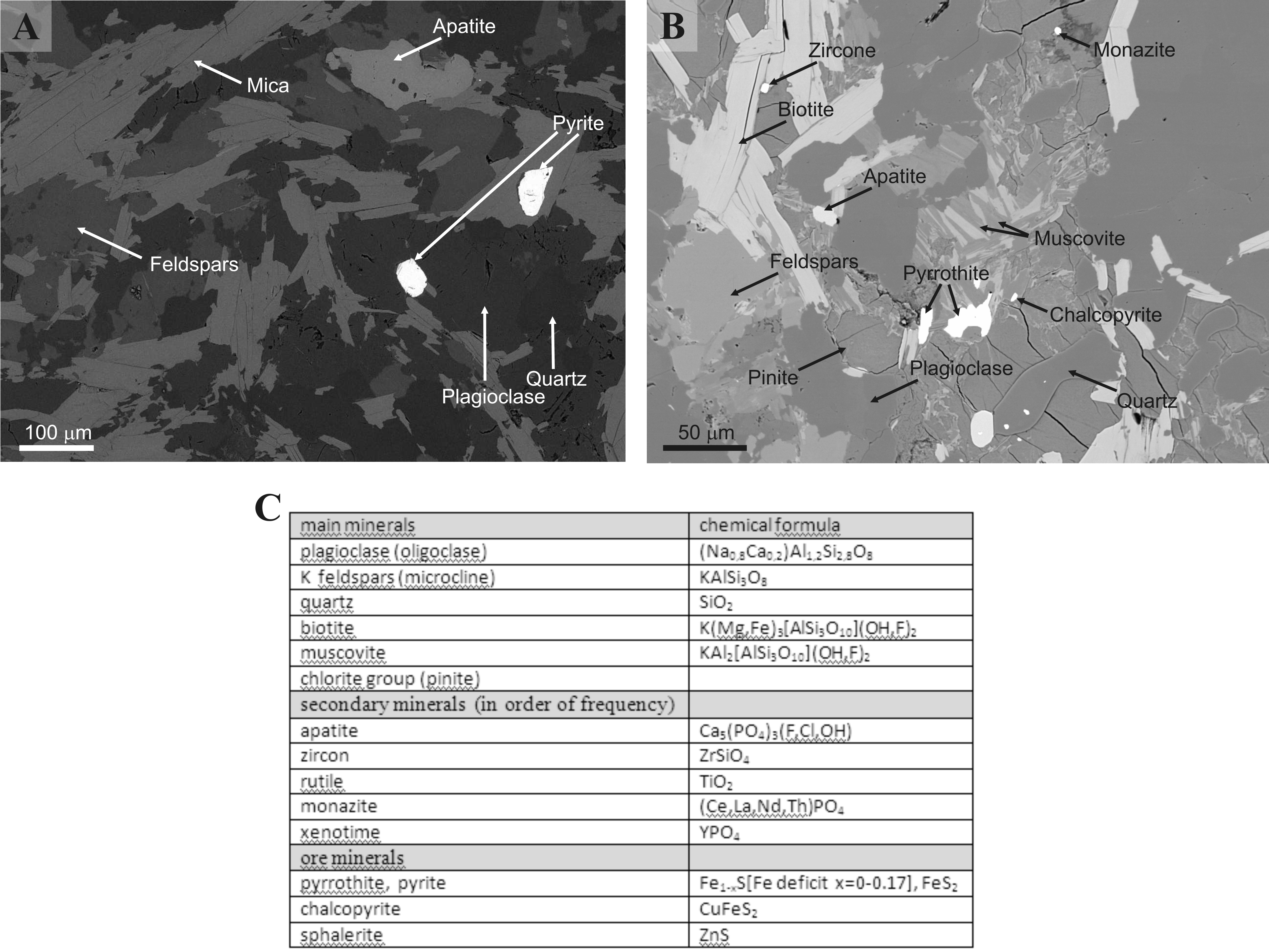

Supplement: Figure S3 [file peerj-05-3635-s003.jpg]

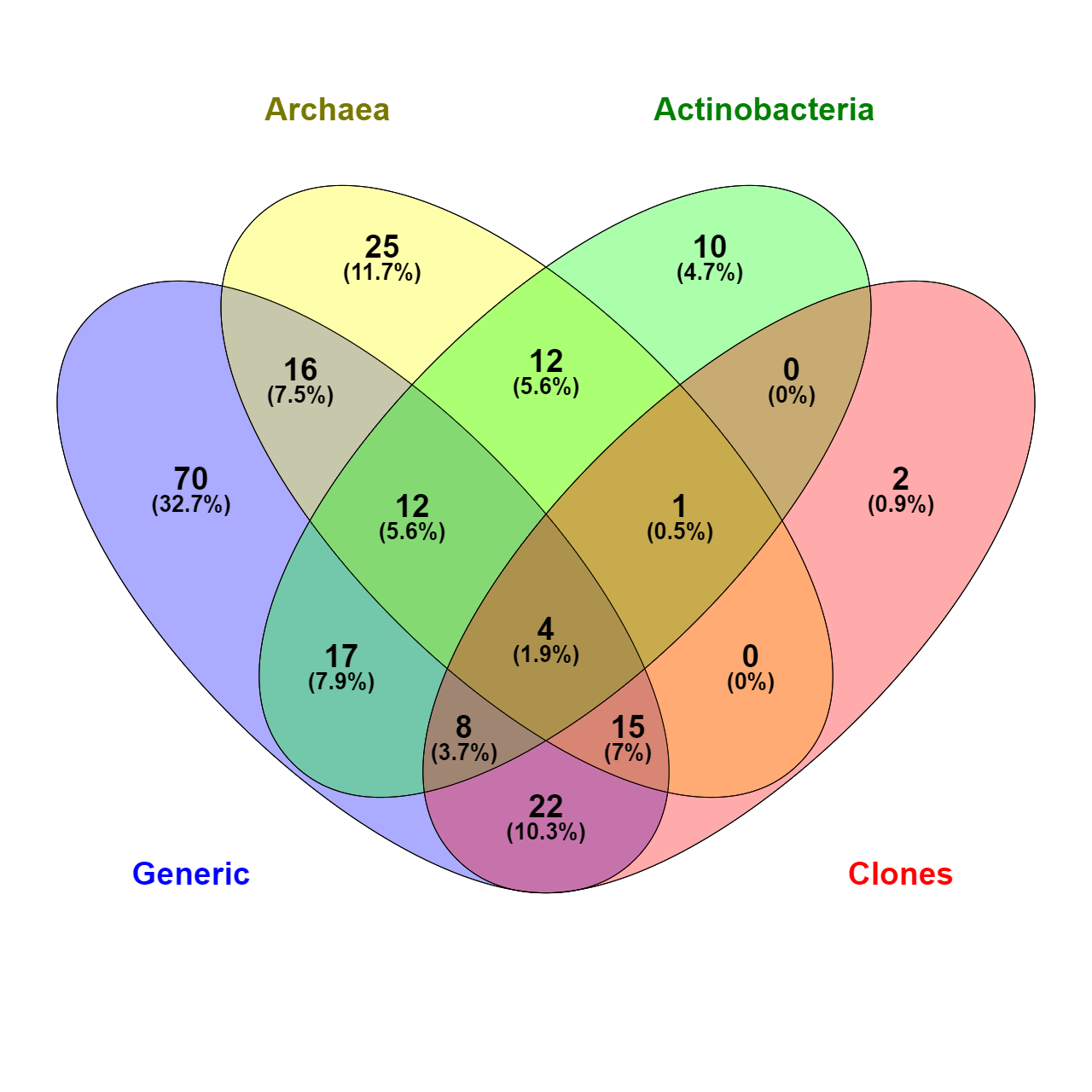

Supplement: Figure S4 [file peerj-05-3635-s004.png]
