# Supplementary material for: Taxonomic and chemical assessment of exceptionally abundant rock mine biofilm
Source: PeerJ. 2017 Aug 15;5:e3635. doi: 10.7717/peerj.3635 (PMC5562143; doi:10.7717/peerj.3635)
Supplement: Table S1 [file peerj-05-3635-s005.docx]

Table S1. PCR primers used in this study.

| **Primer** | **Target 16S rRNA group** | **Sequence 5’ → 3’** | **Reference** |
| --- | --- | --- | --- |
| **27F** | *Bacteria* | AGAGTTTGATCMTGGCTCAG | (Lane, 1991) |
| **357F*** | *Bacteria* | CCTACGGGAGGCAGCAG | (Muyzer *et al*. 1993) |
| **518R*** | Universal | ATTACCGCGGCTGCTGG | (Muyzer *et al*. 1993) |
| **926R** | Universal | CCGTCAATTCCTTTGAGTTT | (Watanabe *et al*. 2001) |
| **M8-A21F** | *Archaea* | CCATCTCATCCCTGCGTGTCTCCGACTCAGCTCGCGTGTCTTCCGGTTGATCCTGCCGG | This work |
| **MB-1204R** | *Archaea* | CCTATCCCCTGTGTGCCTTGGCAGTCTCAGTTCGGGGCATACTGACCT | This work, based on (Baker & Banfield 2003) |
| **MB-518R** | Universal | CCTATCCCCTGTGTGCCTTGGCAGTCTCAGGGTATTACCGCGGCGGCTG | This work |
| **M10-337F** | Universal | CCATCTCATCCCTGCGTGTCTCCGACTCAGTTCCTATGCGGACTCCTACGGGAGGCAGCAG | This work |
| **MB-1159R** | *Actinobacteria* | CCTATCCCCTGTGTGCCTTGGCAGTCTCAGTCCGAGTTCACCCCGGC | This work |
| **M6-16SrR** | *Bacteria* | CCATCTCATCCCTGCGTGTCTCCGACTCAGATATCGCGAGCCATTGTAGCACGTGTGTAGCCC | This work |
| **MB-16SrF** | *Bacteria* | CCTATCCCCTGTGTGCCTTGGCAGTCTCAGAACAGGATTAGATACCCTGGTAGTCCACGC | This work |

* MSSCP; underlined – Multiplex Identifier MID

References

Baker BJ, Banfield JF. (2003). Microbial communities in acid mine drainage. FEMS Microbiol. Ecol. 44:139–152.

Lane DJ. (1991). 16S/23S rRNA sequencing. Nucleic Acid Tech. Bact. Syst. 125–175.

Muyzer G, De Waal EC, Uitterlinden AG. (1993). Profiling of complex microbial populations by denaturing gradient gel electrophoresis analysis of polymerase chain reaction-amplified genes coding for 16S rRNA. Appl. Environ. Microbiol. 59:695–700.

Watanabe K, Kodama Y, Harayama S. (2001). Design and evaluation of PCR primers to amplify bacterial 16S ribosomal DNA fragments used for community fingerprinting. J. Microbiol. Methods 44:253–262.
