# Supplementary material for: Taxonomic and chemical assessment of exceptionally abundant rock mine biofilm
Source: PeerJ. 2017 Aug 15;5:e3635. doi: 10.7717/peerj.3635 (PMC5562143; doi:10.7717/peerj.3635)
Supplement: Table S2 [file peerj-05-3635-s006.docx]

Table S2. PCR conditions.

| **Target** | **Primer** | **Initial denaturation temperature/time** | **Denaturation**  **temperature/ time** | **Annealing temperature/ time** | **Extension**  **temperature/ time** | **Final extension temperature/ time** | **Cycle** |
| --- | --- | --- | --- | --- | --- | --- | --- |
| V3 | 357F  518R | 94 °C/5 min. | 94 °C/20 sec. | 51 °C/45 sec. | 72 °C/1 min. | 72 °C/7 min. | 30 |
| V1-V5 | 27F  926R | 95 °C/3 min. | 95 °C/30 sec. | 53 °C/30 sec. | 72 °C/1 min. | 72 °C/7 min. | 30 |
| V5-V7 | MB-16SrF  M6-16SrR | 95 °C/5 min. | 95 °C/30 sec. | 59 °C/30 sec. | 72 °C/1 min. | 72 °C/7 min. | 30 |
| V3-V7 | M10-337F  MB-1159R | 95 °C/3 min. | 95 °C/30 sec. | 53 °C/30 sec. | 72 °C/1,10 min. | 72 °C/7 min. | 30 |
| V1-V3 | M8-A21F  MB-1204R | 95 °C/3 min. | 95 °C/30 sec. | 65 °C/30 sec. | 72 °C/1,10 min. | 72 °C/7 min. | 30 |
|  | M8-A21F  MB-518R  * | 95 °C/3 min. | 95 °C/30 sec. | 65 °C/30 sec. | 72 °C/40 sec. | 72 °C/7 min. | 30 |

* nested PCR
