# Supplementary material for: Taxonomic and chemical assessment of exceptionally abundant rock mine biofilm
Source: PeerJ. 2017 Aug 15;5:e3635. doi: 10.7717/peerj.3635 (PMC5562143; doi:10.7717/peerj.3635)
Supplement: Table S3 [file peerj-05-3635-s007.pdf]

Taxon	Total	Clones	Act	Generic	Archea
Archaea	2122	0	14	0	2108
Crenarchaeota	376	0	7	0	369
Thermoprotei	376	0	7	0	369
Acidilobales	2	0	2	0	0
Acidilobaceae	2	0	2	0	0
Desulfurococcales	129	0	2	0	127
Desulfurococcaceae	61	0	2	0	59
Pyrodictiaceae	68	0	0	0	68
Sulfolobales	26	0	0	0	26
Sulfolobaceae	26	0	0	0	26
Thermoproteales	219	0	3	0	216
Thermofilaceae	84	0	0	0	84
Thermoproteaceae	135	0	3	0	132
Euryarchaeota	1746	0	7	0	1739
Methanomicrobia	92	0	2	0	90
Methanocellales	22	0	0	0	22
Methanocellaceae	22	0	0	0	22
Methanomicrobiales	63	0	0	0	63
Methanomicrobiaceae	15	0	0	0	15
Methanomicrobiales_incertae_sedis	48	0	0	0	48
Methanosarcinales	7	0	2	0	5
Methanosarcinaceae	6	0	2	0	4
Methermicoccaceae	1	0	0	0	1
Archaeoglobi	58	0	0	0	58
Archaeoglobales	58	0	0	0	58
Archaeoglobaceae	58	0	0	0	58
Halobacteria	580	0	5	0	575
Halobacteriales	580	0	5	0	575
Halobacteriaceae	580	0	5	0	575
Methanococci	20	0	0	0	20
Methanococcales	20	0	0	0	20
Methanocaldococcaceae	20	0	0	0	20
Methanopyri	153	0	0	0	153
Methanopyrales	153	0	0	0	153
Methanopyraceae	153	0	0	0	153
Thermoplasmata	843	0	0	0	843
Thermoplasmatales	843	0	0	0	843
Picrophilaceae	838	0	0	0	838
Thermoplasmatales_incertae_sedis	5	0	0	0	5
Bacteria	26069	329	255	25019	466
Acidobacteria	297	4	0	293	0
Acidobacteria_Gp1	5	0	0	5	0
Acidobacteria_Gp10	27	0	0	27	0
Acidobacteria_Gp21	1	0	0	1	0
Acidobacteria_Gp22	4	0	0	4	0
Acidobacteria_Gp3	143	2	0	141	0
Acidobacteria_Gp4	2	2	0	0	0
Acidobacteria_Gp7	34	0	0	34	0
Holophagae	81	0	0	81	0
Acanthopleuribacterales	48	0	0	48	0
Acanthopleuribacteraceae	48	0	0	48	0
Holophagales	33	0	0	33	0
Holophagaceae	33	0	0	33	0
Actinobacteria	518	1	100	370	47
Actinobacteria	518	1	100	370	47
Acidimicrobiales	34	0	31	2	1
Acidimicrobiaceae	16	0	15	0	1
Iamiaceae	18	0	16	2	0
Actinobacteria_order_incertae_sedis	27	0	23	4	0
Acidimicrobidae_incertae_sedis	27	0	23	4	0
Actinomycetales	219	1	40	176	2
Acidothermaceae	6	0	0	6	0
Actinosynnemataceae	2	0	2	0	0
Catenulisporaceae	2	0	0	2	0
Cellulomonadaceae	10	0	2	8	0
Cryptosporangiaceae	1	0	1	0	0
Dietziaceae	1	0	1	0	0
Frankineae_incertae_sedis	1	0	0	1	0
Geodermatophilaceae	6	0	1	5	0
Glycomycetaceae	1	0	0	1	0
Intrasporangiaceae	1	0	0	1	0
Microbacteriaceae	5	0	0	3	2
Micrococcaceae	2	0	2	0	0
Micromonosporaceae	26	1	13	12	0
Mycobacteriaceae	3	0	3	0	0
Nakamurellaceae	1	0	0	1	0
Nocardiaceae	85	0	1	84	0

Promicromonosporaceae	6	0	0	6	0
Propionibacteriaceae	29	0	3	26	0
Pseudonocardiaceae	22	0	7	15	0
Sporichthyaceae	3	0	2	1	0
Streptomycetaceae	2	0	0	2	0
Streptosporangiaceae	2	0	2	0	0
Thermomonosporaceae	1	0	0	1	0
Tsukamurellaceae	1	0	0	1	0
Bifidobacteriales	16	0	0	0	16
Bifidobacteriaceae	16	0	0	0	16
Coriobacteriales	29	0	4	0	25
Coriobacteriaceae	29	0	4	0	25
Nitriliruptorales	77	0	1	73	3
Nitriliruptoraceae	77	0	1	73	3
Solirubrobacterales	116	0	1	115	0
Conexibacteraceae	96	0	1	95	0
Patulibacteraceae	6	0	0	6	0
Solirubrobacteraceae	14	0	0	14	0
Aquificae	17	0	3	3	11
Aquificae	17	0	3	3	11
Aquificales	17	0	3	3	11
Aquificaceae	5	0	1	0	4
Aquificales_incertae_sedis	4	0	1	3	0
Desulfurobacteriaceae	8	0	1	0	7
Bacteroidetes	1217	12	3	1147	55
Bacteroidetes_class_incertae_sedis	26	0	0	21	5
Bacteroidetes_order_incertae_sedis	26	0	0	21	5
Bacteroidetes_incertae_sedis	26	0	0	21	5
Bacteroidia	25	0	1	1	23
Bacteroidales	25	0	1	1	23
Marinilabiaceae	19	0	1	0	18
Porphyromonadaceae	1	0	0	0	1
Prevotellaceae	3	0	0	0	3
Rikenellaceae	1	0	0	1	0
Bacteroidales_incertae_sedis	1	0	0	0	1
Sphingobacteria	1021	11	0	989	21
Sphingobacteriales	1021	11	0	989	21
Chitinophagaceae	384	4	0	375	5
Cyclobacteriaceae	16	1	0	15	0
Flammeovirgaceae	211	0	0	211	0
Rhodothermaceae	17	0	0	3	14
Saprospiraceae	153	1	0	152	0
Cytophagaceae	206	5	0	200	1
Sphingobacteriaceae	34	0	0	33	1
Flavobacteria	145	1	2	136	6
Flavobacteriales	145	1	2	136	6
Cryomorphaceae	113	1	0	109	3
Flavobacteriaceae	32	0	2	27	3
Chlamydiae	44	0	1	43	0
Chlamydiae	44	0	1	43	0
Chlamydiales	44	0	1	43	0
Parachlamydiaceae	33	0	1	32	0
Simkaniaceae	1	0	0	1	0
Waddliaceae	10	0	0	10	0
Chlorobi	2	0	0	0	2
Chlorobia	2	0	0	0	2
Chlorobiales	2	0	0	0	2
Chlorobiaceae	2	0	0	0	2
Chloroflexi	657	33	8	548	68
Chloroflexi	50	1	5	12	32
Chloroflexales	35	1	5	0	29
Chloroflexaceae	35	1	5	0	29
Herpetosiphonales	15	0	0	12	3
Herpetosiphonaceae	15	0	0	12	3
Anaerolineae	500	16	2	481	1
Anaerolineales	500	16	2	481	1
Anaerolineaceae	500	16	2	481	1
Caldilineae	67	16	0	50	1
Caldilineales	67	16	0	50	1
Caldilineaceae	67	16	0	50	1
Thermomicrobia	40	0	1	5	34
Sphaerobacterales	8	0	0	0	8
Sphaerobacteraceae	8	0	0	0	8
Thermomicrobiales	32	0	1	5	26
Thermomicrobiaceae	32	0	1	5	26
Deferribacteres	24	1	0	23	0
Deferribacteres	24	1	0	23	0
Deferribacterales	24	1	0	23	0

Deferribacteraceae	23	0	0	23	0
Deferribacterales_incertae_sedis	1	1	0	0	0
Deinococcus Thermus	9	0	0	1	8
Deinococci	9	0	0	1	8
Deinococcales	1	0	0	1	0
Trueperaceae	1	0	0	1	0
Thermales	8	0	0	0	8
Thermaceae	8	0	0	0	8
Firmicutes	452	3	66	275	108
Bacilli	209	1	28	172	8
Lactobacillales	185	0	22	156	7
Carnobacteriaceae	4	0	0	0	4
Enterococcaceae	2	0	0	0	2
Leuconostocaceae	156	0	9	147	0
Lactobacillaceae	13	0	12	1	0
Streptococcaceae	10	0	1	8	1
Bacillales	24	1	6	16	1
Listeriaceae	2	0	1	1	0
Sporolactobacillaceae	3	0	3	0	0
Staphylococcaceae	4	0	0	4	0
Bacillaceae	6	1	1	4	0
Planococcaceae	2	0	0	2	0
Thermoactinomycetaceae	7	0	1	5	1
Clostridia	238	2	36	103	97
Thermoanaerobacterales	130	1	5	46	78
Thermoanaerobacteraceae	52	1	5	46	0
Thermodesulfobiaceae	78	0	0	0	78
Clostridiales	102	1	31	51	19
Lachnospiraceae	6	0	1	0	5
Ruminococcaceae	6	0	1	4	1
Clostridiaceae	57	0	28	22	7
Incertae_Sedis_XI	11	0	0	11	0
Incertae_Sedis_XIII	3	0	0	3	0
Incertae_Sedis_XIV	3	0	0	1	2
Incertae_Sedis_XVIII	7	0	0	5	2
Peptococcaceae	2	0	1	0	1
Veillonellaceae	7	1	0	5	1
Natranaerobiales	6	0	0	6	0
Natranaerobiaceae	6	0	0	6	0
Erysipelotrichi	5	0	2	0	3
Erysipelotrichales	5	0	2	0	3
Erysipelotrichaceae	5	0	2	0	3
Fusobacteria	2	0	0	1	1
Fusobacteria	2	0	0	1	1
Fusobacteriales	2	0	0	1	1
Leptotrichiaceae	2	0	0	1	1
Gemmatimonadetes	375	0	0	375	0
Gemmatimonadetes	375	0	0	375	0
Gemmatimonadales	375	0	0	375	0
Gemmatimonadaceae	375	0	0	375	0
Lentisphaerae	3	0	3	0	0
Lentisphaeria	3	0	3	0	0
Lentisphaerales	2	0	2	0	0
Lentisphaeraceae	2	0	2	0	0
Victivallales	1	0	1	0	0
Victivallaceae	1	0	1	0	0
Nitrospira	38	0	1	36	1
Nitrospira	38	0	1	36	1
Nitrospirales	38	0	1	36	1
Nitrospiraceae	38	0	1	36	1
Planctomycetes	147	36	2	73	36
Planctomycetacia	147	36	2	73	36
Planctomycetales	147	36	2	73	36
Planctomycetaceae	147	36	2	73	36
Proteobacteria	22085	207	58	21705	115
Alphaproteobacteria	17571	151	42	17337	41
Parvularculales	333	16	0	317	0
Parvularculaceae	333	16	0	317	0
Alphaproteobacteria_order_incertae_sedis	611	14	0	597	0
Alphaproteobacteria_incertae_sedis	611	14	0	597	0
Caulobacterales	1453	10	0	1443	0
Caulobacteraceae	79	0	0	79	0
Hyphomonadaceae	1374	10	0	1364	0
Kiloniellales	145	2	0	143	0
Kiloniellaceae	145	2	0	143	0
Kordiimonadales	1	0	0	1	0
Kordiimonadaceae	1	0	0	1	0
Rhizobiales	10657	93	2	10543	19

Aurantimonadaceae	83	1	0	65	17
Bartonellaceae	8	0	0	8	0
Beijerinckiaceae	1739	28	2	1709	0
Bradyrhizobiaceae	353	2	0	351	0
Brucellaceae	27	0	0	26	1
Cohaesibacteraceae	13	0	0	13	0
Hyphomicrobiaceae	4174	57	0	4117	0
Methylobacteriaceae	1932	0	0	1932	0
Methylocystaceae	274	1	0	272	1
Phyllobacteriaceae	264	3	0	261	0
Rhizobiaceae	107	0	0	107	0
Rhizobiales_incertae_sedis	4	0	0	4	0
Rhodobiaceae	131	0	0	131	0
Xanthobacteraceae	1548	1	0	1547	0
Rhodobacterales	471	3	1	465	2
Rhodobacteraceae	471	3	1	465	2
Rhodospirillales	3561	12	39	3490	20
Acetobacteraceae	782	0	3	759	20
Rhodospirillaceae	2779	12	36	2731	0
Rickettsiales	6	0	0	6	0
Anaplasmataceae	1	0	0	1	0
Rickettsiaceae	5	0	0	5	0
Sneathiellales	13	0	0	13	0
Sneathiellaceae	13	0	0	13	0
Sphingomonadales	320	1	0	319	0
Erythrobacteraceae	35	0	0	35	0
Sphingomonadaceae	285	1	0	284	0
Betaproteobacteria	164	12	3	143	6
Burkholderiales	75	10	1	60	4
Alcaligenaceae	13	6	0	7	0
Burkholderiaceae	15	0	0	14	1
Burkholderiales_incertae_sedis	18	1	1	16	0
Comamonadaceae	27	1	0	23	3
Oxalobacteraceae	2	2	0	0	0
Hydrogenophilales	22	0	2	19	1
Hydrogenophilaceae	22	0	2	19	1
Neisseriales	56	0	0	56	0
Neisseriaceae	56	0	0	56	0
Rhodocyclales	11	2	0	8	1
Rhodocyclaceae	11	2	0	8	1
Deltaproteobacteria	2873	35	2	2789	47
Bdellovibrionales	25	0	0	25	0
Bacteriovoracaceae	7	0	0	7	0
Bdellovibrionaceae	18	0	0	18	0
Desulfarculales	6	4	0	2	0
Desulfarculaceae	6	4	0	2	0
Desulfobacterales	298	1	0	293	4
Desulfobacteraceae	61	1	0	57	3
Desulfobulbaceae	237	0	0	236	1
Desulfovibrionales	13	4	0	4	5
Desulfohalobiaceae	5	2	0	3	0
Desulfovibrionaceae	8	2	0	1	5
Desulfurellales	77	0	0	77	0
Desulfurellaceae	77	0	0	77	0
Desulfuromonadales	65	9	0	56	0
Desulfuromonadaceae	23	0	0	23	0
Geobacteraceae	42	9	0	33	0
Myxococcales	1766	15	2	1743	6
Haliangiaceae	212	0	0	212	0
Cystobacteraceae	748	1	0	745	2
Kofleriaceae	69	2	0	67	0
Myxococcaceae	40	4	0	36	0
Nannocystaceae	139	2	2	133	2
Phaselicystidaceae	207	1	0	206	0
Polyangiaceae	351	5	0	344	2
Syntrophobacterales	623	2	0	589	32
Syntrophaceae	149	0	0	149	0
Syntrophobacteraceae	474	2	0	440	32
Epsilonproteobacteria	17	0	0	16	1
Campylobacterales	9	0	0	9	0
Hydrogenimonaceae	3	0	0	3	0
Helicobacteraceae	6	0	0	6	0
Nautiliales	8	0	0	7	1
Nautiliaceae	8	0	0	7	1
Gammaproteobacteria	1460	9	11	1420	20
Enterobacteriales	118	2	9	107	0
Enterobacteriaceae	118	2	9	107	0
Salinisphaerales	1	0	0	1	0

Salinisphaeraceae	1	0	0	1	0
Vibrionales	2	0	0	2	0
Vibrionaceae	2	0	0	2	0
Acidithiobacillales	185	0	0	185	0
Thermithiobacillaceae	185	0	0	185	0
Aeromonadales	3	0	0	3	0
Succinivibrionaceae	3	0	0	3	0
Alteromonadales	107	0	0	107	0
Alteromonadaceae	32	0	0	32	0
Ferrimonadaceae	2	0	0	2	0
Moritellaceae	65	0	0	65	0
Pseudoalteromonadaceae	1	0	0	1	0
Shewanellaceae	7	0	0	7	0
Cardiobacteriales	5	0	0	5	0
Cardiobacteriaceae	5	0	0	5	0
Chromatiales	153	2	0	135	16
Chromatiaceae	115	2	0	113	0
Ectothiorhodospiraceae	12	0	0	11	1
Granulosicoccaceae	20	0	0	5	15
Halothiobacillaceae	6	0	0	6	0
Gammaproteobacteria_order_incertae_sedis	114	0	0	114	0
Gammaproteobacteria_incertae_sedis	114	0	0	114	0
Legionellales	135	0	0	135	0
Coxiellaceae	86	0	0	86	0
Legionellaceae	49	0	0	49	0
Methylococcales	75	0	0	75	0
Methylococcaceae	75	0	0	75	0
Oceanospirillales	61	0	1	60	0
Alcanivoracaceae	1	0	0	1	0
Hahellaceae	2	0	0	2	0
Halomonadaceae	15	0	0	15	0
Oceanospirillaceae	39	0	1	38	0
Oceanospirillales_incertae_sedis	4	0	0	4	0
Pasteurellales	9	0	1	8	0
Pasteurellaceae	9	0	1	8	0
Pseudomonadales	57	1	0	56	0
Moraxellaceae	12	0	0	12	0
Pseudomonadaceae	45	1	0	44	0
Thiotrichales	25	3	0	21	1
Piscirickettsiaceae	16	0	0	16	0
Thiotrichaceae	4	1	0	3	0
Thiotrichales_incertae_sedis	5	2	0	2	1
Xanthomonadales	410	1	0	406	3
Sinobacteraceae	289	1	0	288	0
Xanthomonadaceae	121	0	0	118	3
Spirochaetes	8	0	0	8	0
Spirochaetes	8	0	0	8	0
Spirochaetales	8	0	0	8	0
Leptospiraceae	8	0	0	8	0
Synergistetes	15	0	3	7	5
Synergistia	15	0	3	7	5
Synergistales	15	0	3	7	5
Synergistaceae	15	0	3	7	5
Tenericutes	6	0	3	1	2
Mollicutes	6	0	3	1	2
Anaeroplasmatales	6	0	3	1	2
Anaeroplasmataceae	6	0	3	1	2
Thermodesulfobacteria	52	0	3	49	0
Thermodesulfobacteria	52	0	3	49	0
Thermodesulfobacteriales	52	0	3	49	0
Thermodesulfobacteriaceae	52	0	3	49	0
Thermotogae	4	0	0	0	4
Thermotogae	4	0	0	0	4
Thermotogales	4	0	0	0	4
Thermotogaceae	4	0	0	0	4
Verrucomicrobia	78	31	1	44	2
Opitutae	42	0	0	40	2
Opitutales	41	0	0	40	1
Opitutaceae	41	0	0	40	1
Puniceicoccales	1	0	0	0	1
Puniceicoccaceae	1	0	0	0	1
Spartobacteria	23	23	0	0	0
Subdivision3	2	2	0	0	0
Verrucomicrobiae	11	6	1	4	0
Verrucomicrobiales	11	6	1	4	0
Verrucomicrobiaceae	11	6	1	4	0
Bacteria_incertae_sedis	7	0	0	7	0
Ktedonobacteria	7	0	0	7	0

Ktedonobacterales	7	0	0	7	0
Ktedonobacteraceae	7	0	0	7	0
Cyanobacteria	1	0	0	0	1
Cyanobacteria	1	0	0	0	1
Cyanobacteria_order_incertae_sedis	1	0	0	0	1
Chloroplast	1	0	0	0	1
OP10	11	1	0	10	0
