# Supplementary material for: Taxonomic and chemical assessment of exceptionally abundant rock mine biofilm
Source: PeerJ. 2017 Aug 15;5:e3635. doi: 10.7717/peerj.3635 (PMC5562143; doi:10.7717/peerj.3635)
Supplement: Table S4 — Length of colored data stripes is proportional to OTU’s number. (Clones) refers to data obtained from Tomczyk-Żak et al. (2013). [file peerj-05-3635-s008.pdf]

taxl eve	rankID	Taxon	daught	Total	Clones	Act	Generic	Archea
0	0	Root	2	0	0	0	0	0
1	0,1	Archaea	2	38	0	10	0	37
2	0.1.1	Crenarchaeota	1	13	0	5	0	12
3	0.1.1.1	Thermoprotei	4	13	0	5	0	12
4	0.1.1.1.1	Acidilobales	1	1	0	1	0	0
5	0.1.1.1.1.1	Acidilobaceae	1	1	0	1	0	0
6	0.1.1.1.1.1.1	Acidilobus	0	1	0	1	0	0
4	0.1.1.1.2	Desulfurococcales	2	8	0	2	0	8
5	0.1.1.1.2.1	Desulfurococcaceae	5	5	0	2	0	5
6	0.1.1.1.2.1.1	Ignisphaera	0	1	0	0	0	1
6	0.1.1.1.2.1.2	Stetteria	0	1	0	0	0	1
6	0.1.1.1.2.1.3	Sulfophobococcus	0	1	0	0	0	1
6	0.1.1.1.2.1.4	Thermodiscus	0	1	0	1	0	1
6	0.1.1.1.2.1.5	Thermosphaera	0	1	0	1	0	1
5	0.1.1.1.2.2	Pyrodictiaceae	3	3	0	0	0	3
6	0.1.1.1.2.2.1	Hyperthermus	0	1	0	0	0	1
6	0.1.1.1.2.2.2	Pyrodictium	0	1	0	0	0	1
6	0.1.1.1.2.2.3	Pyrolobus	0	1	0	0	0	1
4	0.1.1.1.3	Sulfolobales	1	1	0	0	0	1
5	0.1.1.1.3.1	Sulfolobaceae	1	1	0	0	0	1
6	0.1.1.1.3.1.1	Sulfurisphaera	0	1	0	0	0	1
4	0.1.1.1.4	Thermoproteales	2	3	0	2	0	3
5	0.1.1.1.4.1	Thermofilaceae	1	1	0	0	0	1
6	0.1.1.1.4.1.1	Thermofilum	0	1	0	0	0	1
5	0.1.1.1.4.2	Thermoproteaceae	2	2	0	2	0	2
6	0.1.1.1.4.2.1	Caldivirga	0	1	0	1	0	1
6	0.1.1.1.4.2.2	Thermocladium	0	1	0	1	0	1
2	0.1.2	Euryarchaeota	6	25	0	5	0	25
3	0.1.2.1	Methanomicrobia	3	8	0	1	0	8
4	0.1.2.1.1	Methanocellales	1	1	0	0	0	1
5	0.1.2.1.1.1	Methanocellaceae	1	1	0	0	0	1
6	0.1.2.1.1.1.1	Methanocella	0	1	0	0	0	1
4	0.1.2.1.2	Methanomicrobiales	2	4	0	0	0	4
5	0.1.2.1.2.1	Methanomicrobiaceae	3	3	0	0	0	3
6	0.1.2.1.2.1.1	Methanolacinia	0	1	0	0	0	1
6	0.1.2.1.2.1.2	Methanomicrobium	0	1	0	0	0	1
6	0.1.2.1.2.1.3	Methanosphaerula	0	1	0	0	0	1
5	0.1.2.1.2.2	Methanomicrobiales_incertae_s	1	1	0	0	0	1
6	0.1.2.1.2.2.1	Methanolinea	0	1	0	0	0	1
4	0.1.2.1.3	Methanosarcinales	2	3	0	1	0	3
5	0.1.2.1.3.1	Methanosarcinaceae	2	2	0	1	0	2
6	0.1.2.1.3.1.1	Methanimicrococcus	0	1	0	1	0	1
6	0.1.2.1.3.1.2	Methanosalsum	0	1	0	0	0	1
5	0.1.2.1.3.2	Methermicoccaceae	1	1	0	0	0	1
6	0.1.2.1.3.2.1	Methermicoccus	0	1	0	0	0	1
3	0.1.2.2	Archaeoglobi	1	2	0	0	0	2
4	0.1.2.2.1	Archaeoglobales	1	2	0	0	0	2
5	0.1.2.2.1.1	Archaeoglobaceae	2	2	0	0	0	2
6	0.1.2.2.1.1.1	Ferroglobus	0	1	0	0	0	1
6	0.1.2.2.1.1.2	Geoglobus	0	1	0	0	0	1
3	0.1.2.3	Halobacteria	1	11	0	4	0	11
4	0.1.2.3.1	Halobacteriales	1	11	0	4	0	11
5	0.1.2.3.1.1	Halobacteriaceae	11	11	0	4	0	11
6	0.1.2.3.1.1.1	Haladaptatus	0	1	0	0	0	1
6	0.1.2.3.1.1.2	Halogeometricum	0	1	0	0	0	1
6	0.1.2.3.1.1.3	Halomicrobium	0	1	0	0	0	1
6	0.1.2.3.1.1.4	Halopiger	0	1	0	0	0	1
6	0.1.2.3.1.1.5	Haloplanus	0	1	0	1	0	1
6	0.1.2.3.1.1.6	Haloquadratum	0	1	0	1	0	1
6	0.1.2.3.1.1.7	Halosarcina	0	1	0	1	0	1
6	0.1.2.3.1.1.8	Halosimplex	0	1	0	0	0	1
6	0.1.2.3.1.1.9	Halostagnicola	0	1	0	1	0	1
6	0.1.2.3.1.1.10	Natronobacterium	0	1	0	0	0	1
6	0.1.2.3.1.1.11	Natronomonas	0	1	0	0	0	1
3	0.1.2.4	Methanococci	1	1	0	0	0	1
4	0.1.2.4.1	Methanococcales	1	1	0	0	0	1
5	0.1.2.4.1.1	Methanocaldococcaceae	1	1	0	0	0	1
6	0.1.2.4.1.1.1	Methanotorris	0	1	0	0	0	1
3	0.1.2.5	Methanopyri	1	1	0	0	0	1
4	0.1.2.5.1	Methanopyrales	1	1	0	0	0	1
5	0.1.2.5.1.1	Methanopyraceae	1	1	0	0	0	1
6	0.1.2.5.1.1.1	Methanopyrus	0	1	0	0	0	1
3	0.1.2.6	Thermoplasmata	1	2	0	0	0	2
4	0.1.2.6.1	Thermoplasmatales	2	2	0	0	0	2
5	0.1.2.6.1.1	Picrophilaceae	1	1	0	0	0	1
6	0.1.2.6.1.1.1	Picrophilus	0	1	0	0	0	1
5	0.1.2.6.1.2	Thermoplasmatales_incertae_se	1	1	0	0	0	1
6	0.1.2.6.1.2.1	Thermogymnomonas	0	1	0	0	0	1
1	0,2	Bacteria	25	633	93	82	519	104
2	0.2.1	Acidobacteria	8	10	2	0	9	0
3	0.2.1.1	Acidobacteria_Gp1	1	1	0	0	1	0
4	0.2.1.1.1	unclassified	1	1	0	0	1	0
5	0.2.1.1.1.1	unclassified	1	1	0	0	1	0
6	0.2.1.1.1.1.1	unclassified	0	1	0	0	1	0
3	0.2.1.2	Acidobacteria_Gp10	1	1	0	0	1	0
4	0.2.1.2.1	unclassified	1	1	0	0	1	0

5	0.2.1.2.1.1	unclassified	1	1	0	0	1	0
6	0.2.1.2.1.1.1	unclassified	0	1	0	0	1	0
3	0.2.1.3	Acidobacteria_Gp21	1	1	0	0	1	0
4	0.2.1.3.1	unclassified	1	1	0	0	1	0
5	0.2.1.3.1.1	unclassified	1	1	0	0	1	0
6	0.2.1.3.1.1.1	unclassified	0	1	0	0	1	0
3	0.2.1.4	Acidobacteria_Gp22	1	1	0	0	1	0
4	0.2.1.4.1	unclassified	1	1	0	0	1	0
5	0.2.1.4.1.1	unclassified	1	1	0	0	1	0
6	0.2.1.4.1.1.1	unclassified	0	1	0	0	1	0
3	0.2.1.5	Acidobacteria_Gp3	1	1	1	0	1	0
4	0.2.1.5.1	unclassified	1	1	1	0	1	0
5	0.2.1.5.1.1	unclassified	1	1	1	0	1	0
6	0.2.1.5.1.1.1	unclassified	0	1	1	0	1	0
3	0.2.1.6	Acidobacteria_Gp4	1	1	1	0	0	0
4	0.2.1.6.1	unclassified	1	1	1	0	0	0
5	0.2.1.6.1.1	unclassified	1	1	1	0	0	0
6	0.2.1.6.1.1.1	unclassified	0	1	1	0	0	0
3	0.2.1.7	Acidobacteria_Gp7	1	1	0	0	1	0
4	0.2.1.7.1	unclassified	1	1	0	0	1	0
5	0.2.1.7.1.1	unclassified	1	1	0	0	1	0
6	0.2.1.7.1.1.1	unclassified	0	1	0	0	1	0
3	0.2.1.8	Holophagae	2	3	0	0	3	0
4	0.2.1.8.1	Acanthopleuribacterales	1	1	0	0	1	0
5	0.2.1.8.1.1	Acanthopleuribacteraceae	1	1	0	0	1	0
6	0.2.1.8.1.1.1	Acanthopleuribacter	0	1	0	0	1	0
4	0.2.1.8.2	Holophagales	1	2	0	0	2	0
5	0.2.1.8.2.1	Holophagaceae	2	2	0	0	2	0
6	0.2.1.8.2.1.1	Geothrix	0	1	0	0	1	0
6	0.2.1.8.2.1.2	Holophaga	0	1	0	0	1	0
2	0.2.2	Actinobacteria	1	67	1	30	39	13
3	0.2.2.1	Actinobacteria	7	67	1	30	39	13
4	0.2.2.1.1	Acidimicrobiales	2	3	0	2	1	1
5	0.2.2.1.1.1	Acidimicrobiaceae	2	2	0	1	0	1
6	0.2.2.1.1.1.1	Acidimicrobium	0	1	0	0	0	1
6	0.2.2.1.1.1.2	Ferrithrix	0	1	0	1	0	0
5	0.2.2.1.1.2	Iamiaceae	1	1	0	1	1	0
6	0.2.2.1.1.2.1	Iamia	0	1	0	1	1	0
4	0.2.2.1.2	Actinobacteria_order_incertae_	1	1	0	1	1	0
5	0.2.2.1.2.1	Acidimicrobidae_incertae_sedis	1	1	0	1	1	0
6	0.2.2.1.2.1.1	Ilumatobacter	0	1	0	1	1	0
4	0.2.2.1.3	Actinomycetales	24	50	1	23	33	2
5	0.2.2.1.3.1	Acidothermaceae	1	1	0	0	1	0
6	0.2.2.1.3.1.1	Acidothermus	0	1	0	0	1	0
5	0.2.2.1.3.2	Actinosynnemataceae	1	1	0	1	0	0
6	0.2.2.1.3.2.1	Umezawaea	0	1	0	1	0	0
5	0.2.2.1.3.3	Catenulisporaceae	1	1	0	0	1	0
6	0.2.2.1.3.3.1	Catenulispora	0	1	0	0	1	0
5	0.2.2.1.3.4	Cellulomonadaceae	1	1	0	1	1	0
6	0.2.2.1.3.4.1	Actinotalea	0	1	0	1	1	0
5	0.2.2.1.3.5	Cryptosporangiaceae	1	1	0	1	0	0
6	0.2.2.1.3.5.1	Cryptosporangium	0	1	0	1	0	0
5	0.2.2.1.3.6	Dietziaceae	1	1	0	1	0	0
6	0.2.2.1.3.6.1	Dietzia	0	1	0	1	0	0
5	0.2.2.1.3.7	Frankineae_incertae_sedis	1	1	0	0	1	0
6	0.2.2.1.3.7.1	Fodinicola	0	1	0	0	1	0
5	0.2.2.1.3.8	Geodermatophilaceae	1	1	0	1	1	0
6	0.2.2.1.3.8.1	Blastococcus	0	1	0	1	1	0
5	0.2.2.1.3.9	Glycomycetaceae	1	1	0	0	1	0
6	0.2.2.1.3.9.1	Haloglycomyces	0	1	0	0	1	0
5	0.2.2.1.3.10	Intrasporangiaceae	1	1	0	0	1	0
6	0.2.2.1.3.10.1	Lapillicoccus	0	1	0	0	1	0
5	0.2.2.1.3.11	Microbacteriaceae	3	3	0	0	1	2
6	0.2.2.1.3.11.1	Glaciibacter	0	1	0	0	0	1
6	0.2.2.1.3.11.2	Leifsonia	0	1	0	0	1	0
6	0.2.2.1.3.11.3	Okibacterium	0	1	0	0	0	1
5	0.2.2.1.3.12	Micrococcaceae	2	2	0	2	0	0
6	0.2.2.1.3.12.1	Micrococcus	0	1	0	1	0	0
6	0.2.2.1.3.12.2	Rothia	0	1	0	1	0	0
5	0.2.2.1.3.13	Micromonosporaceae	11	11	1	7	8	0
6	0.2.2.1.3.13.1	Actinoplanes	0	1	0	1	0	0
6	0.2.2.1.3.13.2	Asanoa	0	1	0	0	1	0
6	0.2.2.1.3.13.3	Catellatospora	0	1	0	1	1	0
6	0.2.2.1.3.13.4	Dactylosporangium	0	1	0	1	0	0
6	0.2.2.1.3.13.5	Krasilnikovia	0	1	0	0	1	0
6	0.2.2.1.3.13.6	Longispora	0	1	0	1	1	0
6	0.2.2.1.3.13.7	Luedemannella	0	1	1	1	1	0
6	0.2.2.1.3.13.8	Pilimelia	0	1	0	1	1	0
6	0.2.2.1.3.13.9	Planosporangium	0	1	0	0	1	0
6	0.2.2.1.3.13.1	Spirilliplanes	0	1	0	1	0	0
6	0.2.2.1.3.13.1	Verrucosispora	0	1	0	0	1	0
5	0.2.2.1.3.14	Mycobacteriaceae	1	1	0	1	0	0
6	0.2.2.1.3.14.1	Mycobacterium	0	1	0	1	0	0
5	0.2.2.1.3.15	Nakamurellaceae	1	1	0	0	1	0
6	0.2.2.1.3.15.1	Humicoccus	0	1	0	0	1	0
5	0.2.2.1.3.16	Nocardiaceae	3	3	0	1	2	0
6	0.2.2.1.3.16.1	Millisia	0	1	0	0	1	0

6	0.2.2.1.3.16.2	Rhodococcus	0	1	0	1	0	0
6	0.2.2.1.3.16.3	Smaragdicoccus	0	1	0	0	1	0
5	0.2.2.1.3.17	Promicromonosporaceae	1	1	0	0	1	0
6	0.2.2.1.3.17.1	Xylanimicrobium	0	1	0	0	1	0
5	0.2.2.1.3.18	Propionibacteriaceae	5	5	0	1	4	0
6	0.2.2.1.3.18.1	Aestuariimicrobium	0	1	0	0	1	0
6	0.2.2.1.3.18.2	Brooklawnia	0	1	0	0	1	0
6	0.2.2.1.3.18.3	Granulicoccus	0	1	0	1	0	0
6	0.2.2.1.3.18.4	Propionibacterium	0	1	0	0	1	0
6	0.2.2.1.3.18.5	Propionicicella	0	1	0	0	1	0
5	0.2.2.1.3.19	Pseudonocardiaceae	7	7	0	4	4	0
6	0.2.2.1.3.19.1	Actinomycetospora	0	1	0	0	1	0
6	0.2.2.1.3.19.2	Allokutzneria	0	1	0	1	0	0
6	0.2.2.1.3.19.3	Kibdelosporangium	0	1	0	0	1	0
6	0.2.2.1.3.19.4	Pseudonocardia	0	1	0	1	1	0
6	0.2.2.1.3.19.5	Streptoalloteichus	0	1	0	1	0	0
6	0.2.2.1.3.19.6	Thermobispora	0	1	0	1	0	0
6	0.2.2.1.3.19.7	Thermocrispum	0	1	0	0	1	0
5	0.2.2.1.3.20	Sporichthyaceae	1	1	0	1	1	0
6	0.2.2.1.3.20.1	Sporichthya	0	1	0	1	1	0
5	0.2.2.1.3.21	Streptomycetaceae	2	2	0	0	2	0
6	0.2.2.1.3.21.1	Kitasatospora	0	1	0	0	1	0
6	0.2.2.1.3.21.2	Streptomyces	0	1	0	0	1	0
5	0.2.2.1.3.22	Streptosporangiaceae	1	1	0	1	0	0
6	0.2.2.1.3.22.1	Planomonospora	0	1	0	1	0	0
5	0.2.2.1.3.23	Thermomonosporaceae	1	1	0	0	1	0
6	0.2.2.1.3.23.1	Spirillospora	0	1	0	0	1	0
5	0.2.2.1.3.24	Tsukamurellaceae	1	1	0	0	1	0
6	0.2.2.1.3.24.1	Tsukamurella	0	1	0	0	1	0
4	0.2.2.1.4	Bifidobacteriales	1	2	0	0	0	2
5	0.2.2.1.4.1	Bifidobacteriaceae	2	2	0	0	0	2
6	0.2.2.1.4.1.1	Aeriscardovia	0	1	0	0	0	1
6	0.2.2.1.4.1.2	Scardovia	0	1	0	0	0	1
4	0.2.2.1.5	Coriobacteriales	1	7	0	2	0	7
5	0.2.2.1.5.1	Coriobacteriaceae	7	7	0	2	0	7
6	0.2.2.1.5.1.1	Adlercreutzia	0	1	0	0	0	1
6	0.2.2.1.5.1.2	Asaccharobacter	0	1	0	0	0	1
6	0.2.2.1.5.1.3	Coriobacterium	0	1	0	1	0	1
6	0.2.2.1.5.1.4	Cryptobacterium	0	1	0	0	0	1
6	0.2.2.1.5.1.5	Enterorhabdus	0	1	0	0	0	1
6	0.2.2.1.5.1.6	Gordonibacter	0	1	0	0	0	1
6	0.2.2.1.5.1.7	Paraeggerthella	0	1	0	1	0	1
4	0.2.2.1.6	Nitriliruptorales	1	1	0	1	1	1
5	0.2.2.1.6.1	Nitriliruptoraceae	1	1	0	1	1	1
6	0.2.2.1.6.1.1	Nitriliruptor	0	1	0	1	1	1
4	0.2.2.1.7	Solirubrobacterales	3	3	0	1	3	0
5	0.2.2.1.7.1	Conexibacteraceae	1	1	0	1	1	0
6	0.2.2.1.7.1.1	Conexibacter	0	1	0	1	1	0
5	0.2.2.1.7.2	Patulibacteraceae	1	1	0	0	1	0
6	0.2.2.1.7.2.1	Patulibacter	0	1	0	0	1	0
5	0.2.2.1.7.3	Solirubrobacteraceae	1	1	0	0	1	0
6	0.2.2.1.7.3.1	Solirubrobacter	0	1	0	0	1	0
2	0.2.3	Aquificae	1	4	0	3	1	2
3	0.2.3.1	Aquificae	1	4	0	3	1	2
4	0.2.3.1.1	Aquificales	3	4	0	3	1	2
5	0.2.3.1.1.1	Aquificaceae	2	2	0	1	0	1
6	0.2.3.1.1.1.1	Aquifex	0	1	0	0	0	1
6	0.2.3.1.1.1.2	Hydrogenobaculum	0	1	0	1	0	0
5	0.2.3.1.1.2	Aquificales_incertae_sedis	1	1	0	1	1	0
6	0.2.3.1.1.2.1	Thermosulfidibacter	0	1	0	1	1	0
5	0.2.3.1.1.3	Desulfurobacteriaceae	1	1	0	1	0	1
6	0.2.3.1.1.3.1	Balnearium	0	1	0	1	0	1
2	0.2.4	Bacteroidetes	4	70	7	3	58	18
3	0.2.4.1	Bacteroidetes_class_incertae_se	1	1	0	0	1	1
4	0.2.4.1.1	Bacteroidetes_order_incertae_s	1	1	0	0	1	1
5	0.2.4.1.1.1	Bacteroidetes_incertae_sedis	1	1	0	0	1	1
6	0.2.4.1.1.1.1	Fulvivirga	0	1	0	0	1	1
3	0.2.4.2	Bacteroidia	1	6	0	1	1	5
4	0.2.4.2.1	Bacteroidales	5	6	0	1	1	5
5	0.2.4.2.1.1	Marinilabiaceae	1	1	0	1	0	1
6	0.2.4.2.1.1.1	Anaerophaga	0	1	0	1	0	1
5	0.2.4.2.1.2	Porphyromonadaceae	1	1	0	0	0	1
6	0.2.4.2.1.2.1	Petrimonas	0	1	0	0	0	1
5	0.2.4.2.1.3	Prevotellaceae	2	2	0	0	0	2
6	0.2.4.2.1.3.1	Hallella	0	1	0	0	0	1
6	0.2.4.2.1.3.2	Xylanibacter	0	1	0	0	0	1
5	0.2.4.2.1.4	Rikenellaceae	1	1	0	0	1	0
6	0.2.4.2.1.4.1	Rikenella	0	1	0	0	1	0
5	0.2.4.2.1.5	Bacteroidales_incertae_sedis	1	1	0	0	0	1
6	0.2.4.2.1.5.1	Phocaeicola	0	1	0	0	0	1
3	0.2.4.3	Sphingobacteria	1	49	6	0	46	7
4	0.2.4.3.1	Sphingobacteriales	7	49	6	0	46	7
5	0.2.4.3.1.1	Chitinophagaceae	13	13	2	0	13	2
6	0.2.4.3.1.1.1	Balneola	0	1	0	0	1	0
6	0.2.4.3.1.1.2	Chitinophaga	0	1	0	0	1	0
6	0.2.4.3.1.1.3	Ferruginibacter	0	1	0	0	1	0
6	0.2.4.3.1.1.4	Filimonas	0	1	0	0	1	0

6	0.2.4.3.1.1.5	Flavisolibacter	0	1	0	0	1	0
6	0.2.4.3.1.1.6	Gracilimonas	0	1	0	0	1	1
6	0.2.4.3.1.1.7	Lacibacter	0	1	0	0	1	0
6	0.2.4.3.1.1.8	Niabella	0	1	0	0	1	0
6	0.2.4.3.1.1.9	Niastella	0	1	1	0	1	0
6	0.2.4.3.1.1.10	Parasegetibacter	0	1	0	0	1	0
6	0.2.4.3.1.1.11	Sediminibacterium	0	1	0	0	1	1
6	0.2.4.3.1.1.12	Segetibacter	0	1	0	0	1	0
6	0.2.4.3.1.1.13	Terrimonas	0	1	1	0	1	0
5	0.2.4.3.1.2	Cyclobacteriaceae	3	3	1	0	2	0
6	0.2.4.3.1.2.1	Aquiflexum	0	1	1	0	0	0
6	0.2.4.3.1.2.2	Belliella	0	1	0	0	1	0
6	0.2.4.3.1.2.3	Cyclobacterium	0	1	0	0	1	0
5	0.2.4.3.1.3	Flammeovirgaceae	9	9	0	0	9	0
6	0.2.4.3.1.3.1	Fabibacter	0	1	0	0	1	0
6	0.2.4.3.1.3.2	Flexithrix	0	1	0	0	1	0
6	0.2.4.3.1.3.3	Limibacter	0	1	0	0	1	0
6	0.2.4.3.1.3.4	Marinoscillum	0	1	0	0	1	0
6	0.2.4.3.1.3.5	Perexilibacter	0	1	0	0	1	0
6	0.2.4.3.1.3.6	Persicobacter	0	1	0	0	1	0
6	0.2.4.3.1.3.7	Rapidithrix	0	1	0	0	1	0
6	0.2.4.3.1.3.8	Reichenbachiella	0	1	0	0	1	0
6	0.2.4.3.1.3.9	Roseivirga	0	1	0	0	1	0
5	0.2.4.3.1.4	Rhodothermaceae	3	3	0	0	2	3
6	0.2.4.3.1.4.1	Rhodothermus	0	1	0	0	1	1
6	0.2.4.3.1.4.2	Salinibacter	0	1	0	0	0	1
6	0.2.4.3.1.4.3	Salisaeta	0	1	0	0	1	1
5	0.2.4.3.1.5	Saprospiraceae	2	2	1	0	2	0
6	0.2.4.3.1.5.1	Aureispira	0	1	0	0	1	0
6	0.2.4.3.1.5.2	Haliscomenobacter	0	1	1	0	1	0
5	0.2.4.3.1.6	Cytophagaceae	14	14	2	0	14	1
6	0.2.4.3.1.6.1	Arcicella	0	1	0	0	1	0
6	0.2.4.3.1.6.2	Cytophaga	0	1	0	0	1	0
6	0.2.4.3.1.6.3	Dyadobacter	0	1	0	0	1	0
6	0.2.4.3.1.6.4	Effluviibacter	0	1	0	0	1	0
6	0.2.4.3.1.6.5	Emticicia	0	1	0	0	1	0
6	0.2.4.3.1.6.6	Flexibacter	0	1	0	0	1	0
6	0.2.4.3.1.6.7	Hymenobacter	0	1	1	0	1	0
6	0.2.4.3.1.6.8	Larkinella	0	1	0	0	1	0
6	0.2.4.3.1.6.9	Leadbetterella	0	1	0	0	1	0
6	0.2.4.3.1.6.10	Microscilla	0	1	0	0	1	0
6	0.2.4.3.1.6.11	Pontibacter	0	1	0	0	1	0
6	0.2.4.3.1.6.12	Rhodonellum	0	1	0	0	1	0
6	0.2.4.3.1.6.13	Rudanella	0	1	0	0	1	0
6	0.2.4.3.1.6.14	Sporocytophaga	0	1	1	0	1	1
5	0.2.4.3.1.7	Sphingobacteriaceae	5	5	0	0	4	1
6	0.2.4.3.1.7.1	Mucilaginibacter	0	1	0	0	1	0
6	0.2.4.3.1.7.2	Nubsella	0	1	0	0	1	0
6	0.2.4.3.1.7.3	Pedobacter	0	1	0	0	1	0
6	0.2.4.3.1.7.4	Pseudosphingobacterium	0	1	0	0	0	1
6	0.2.4.3.1.7.5	Solitalea	0	1	0	0	1	0
3	0.2.4.4	Flavobacteria	1	14	1	2	10	5
4	0.2.4.4.1	Flavobacteriales	2	14	1	2	10	5
5	0.2.4.4.1.1	Cryomorphaceae	6	6	1	0	5	2
6	0.2.4.4.1.1.1	Brumimicrobium	0	1	0	0	1	0
6	0.2.4.4.1.1.2	Crocinitomix	0	1	0	0	1	0
6	0.2.4.4.1.1.3	Cryomorpha	0	1	0	0	1	0
6	0.2.4.4.1.1.4	Fluviicola	0	1	0	0	1	1
6	0.2.4.4.1.1.5	Lishizhenia	0	1	1	0	0	1
6	0.2.4.4.1.1.6	Owenweeksia	0	1	0	0	1	0
5	0.2.4.4.1.2	Flavobacteriaceae	8	8	0	2	5	3
6	0.2.4.4.1.2.1	Coenonia	0	1	0	0	1	0
6	0.2.4.4.1.2.2	Costertonia	0	1	0	1	0	1
6	0.2.4.4.1.2.3	Flagellimonas	0	1	0	0	0	1
6	0.2.4.4.1.2.4	Flavobacterium	0	1	0	0	1	0
6	0.2.4.4.1.2.5	Lutimonas	0	1	0	0	1	0
6	0.2.4.4.1.2.6	Robiginitalea	0	1	0	1	1	0
6	0.2.4.4.1.2.7	Sandarakinotalea	0	1	0	0	1	0
6	0.2.4.4.1.2.8	Weeksella	0	1	0	0	0	1
2	0.2.5	Chlamydiae	1	4	0	1	4	0
3	0.2.5.1	Chlamydiae	1	4	0	1	4	0
4	0.2.5.1.1	Chlamydiales	3	4	0	1	4	0
5	0.2.5.1.1.1	Parachlamydiaceae	2	2	0	1	2	0
6	0.2.5.1.1.1.1	Neochlamydia	0	1	0	1	1	0
6	0.2.5.1.1.1.2	Parachlamydia	0	1	0	0	1	0
5	0.2.5.1.1.2	Simkaniaceae	1	1	0	0	1	0
6	0.2.5.1.1.2.1	Simkania	0	1	0	0	1	0
5	0.2.5.1.1.3	Waddliaceae	1	1	0	0	1	0
6	0.2.5.1.1.3.1	Waddlia	0	1	0	0	1	0
2	0.2.6	Chlorobi	1	2	0	0	0	2
3	0.2.6.1	Chlorobia	1	2	0	0	0	2
4	0.2.6.1.1	Chlorobiales	1	2	0	0	0	2
5	0.2.6.1.1.1	Chlorobiaceae	2	2	0	0	0	2
6	0.2.6.1.1.1.1	Chloroherpeton	0	1	0	0	0	1
6	0.2.6.1.1.1.2	Prosthecochloris	0	1	0	0	0	1
2	0.2.7	Chloroflexi	4	9	5	3	7	6
3	0.2.7.1	Chloroflexi	2	2	1	1	1	2

4	0.2.7.1.1	Chloroflexales	1	1	1	1	0	1
5	0.2.7.1.1.1	Chloroflexaceae	1	1	1	1	0	1
6	0.2.7.1.1.1.1	Chloroflexus	0	1	1	1	0	1
4	0.2.7.1.2	Herpetosiphonales	1	1	0	0	1	1
5	0.2.7.1.2.1	Herpetosiphonaceae	1	1	0	0	1	1
6	0.2.7.1.2.1.1	Herpetosiphon	0	1	0	0	1	1
3	0.2.7.2	Anaerolineae	1	4	3	1	4	1
4	0.2.7.2.1	Anaerolineales	1	4	3	1	4	1
5	0.2.7.2.1.1	Anaerolineaceae	4	4	3	1	4	1
6	0.2.7.2.1.1.1	Bellilinea	0	1	0	0	1	0
6	0.2.7.2.1.1.2	Leptolinea	0	1	1	1	1	0
6	0.2.7.2.1.1.3	Levilinea	0	1	1	0	1	0
6	0.2.7.2.1.1.4	Longilinea	0	1	1	0	1	1
3	0.2.7.3	Caldilineae	1	1	1	0	1	1
4	0.2.7.3.1	Caldilineales	1	1	1	0	1	1
5	0.2.7.3.1.1	Caldilineaceae	1	1	1	0	1	1
6	0.2.7.3.1.1.1	Caldilinea	0	1	1	0	1	1
3	0.2.7.4	Thermomicrobia	2	2	0	1	1	2
4	0.2.7.4.1	Sphaerobacterales	1	1	0	0	0	1
5	0.2.7.4.1.1	Sphaerobacteraceae	1	1	0	0	0	1
6	0.2.7.4.1.1.1	Sphaerobacter	0	1	0	0	0	1
4	0.2.7.4.2	Thermomicrobiales	1	1	0	1	1	1
5	0.2.7.4.2.1	Thermomicrobiaceae	1	1	0	1	1	1
6	0.2.7.4.2.1.1	Thermomicrobium	0	1	0	1	1	1
2	0.2.8	Deferribacteres	1	5	1	0	4	0
3	0.2.8.1	Deferribacteres	1	5	1	0	4	0
4	0.2.8.1.1	Deferribacterales	2	5	1	0	4	0
5	0.2.8.1.1.1	Deferribacteraceae	4	4	0	0	4	0
6	0.2.8.1.1.1.1	Calditerrivibrio	0	1	0	0	1	0
6	0.2.8.1.1.1.2	Denitrovibrio	0	1	0	0	1	0
6	0.2.8.1.1.1.3	Flexistipes	0	1	0	0	1	0
6	0.2.8.1.1.1.4	Mucispirillum	0	1	0	0	1	0
5	0.2.8.1.1.2	Deferribacterales_incertae_sedi	1	1	1	0	0	0
6	0.2.8.1.1.2.1	Caldithrix	0	1	1	0	0	0
2	0.2.9	Deinococcus-Thermus	1	3	0	0	1	2
3	0.2.9.1	Deinococci	2	3	0	0	1	2
4	0.2.9.1.1	Deinococcales	1	1	0	0	1	0
5	0.2.9.1.1.1	Trueperaceae	1	1	0	0	1	0
6	0.2.9.1.1.1.1	Truepera	0	1	0	0	1	0
4	0.2.9.1.2	Thermales	1	2	0	0	0	2
5	0.2.9.1.2.1	Thermaceae	2	2	0	0	0	2
6	0.2.9.1.2.1.1	Marinithermus	0	1	0	0	0	1
6	0.2.9.1.2.1.2	Vulcanithermus	0	1	0	0	0	1
2	0.2.10	Firmicutes	3	55	3	17	30	16
3	0.2.10.1	Bacilli	2	18	1	8	12	5
4	0.2.10.1.1	Lactobacillales	5	9	0	4	5	4
5	0.2.10.1.1.1	Carnobacteriaceae	2	2	0	0	0	2
6	0.2.10.1.1.1.1	Atopococcus	0	1	0	0	0	1
6	0.2.10.1.1.1.2	Dolosigranulum	0	1	0	0	0	1
5	0.2.10.1.1.2	Enterococcaceae	1	1	0	0	0	1
6	0.2.10.1.1.2.1	Pilibacter	0	1	0	0	0	1
5	0.2.10.1.1.3	Leuconostocaceae	1	1	0	1	1	0
6	0.2.10.1.1.3.1	Leuconostoc	0	1	0	1	1	0
5	0.2.10.1.1.4	Lactobacillaceae	2	2	0	2	1	0
6	0.2.10.1.1.4.1	Lactobacillus	0	1	0	1	1	0
6	0.2.10.1.1.4.2	Paralactobacillus	0	1	0	1	0	0
5	0.2.10.1.1.5	Streptococcaceae	3	3	0	1	3	1
6	0.2.10.1.1.5.1	Lactococcus	0	1	0	1	1	0
6	0.2.10.1.1.5.2	Lactovum	0	1	0	0	1	1
6	0.2.10.1.1.5.3	Streptococcus	0	1	0	0	1	0
4	0.2.10.1.2	Bacillales	6	9	1	4	7	1
5	0.2.10.1.2.1	Listeriaceae	1	1	0	1	1	0
6	0.2.10.1.2.1.1	Brochothrix	0	1	0	1	1	0
5	0.2.10.1.2.2	Sporolactobacillaceae	1	1	0	1	0	0
6	0.2.10.1.2.2.1	Sporolactobacillus	0	1	0	1	0	0
5	0.2.10.1.2.3	Staphylococcaceae	2	2	0	0	2	0
6	0.2.10.1.2.3.1	Gemella	0	1	0	0	1	0
6	0.2.10.1.2.3.2	Staphylococcus	0	1	0	0	1	0
5	0.2.10.1.2.4	Bacillaceae	3	3	1	1	2	0
6	0.2.10.1.2.4.1	Bacillus	0	1	1	0	1	0
6	0.2.10.1.2.4.2	Saccharococcus	0	1	0	1	0	0
6	0.2.10.1.2.4.3	Virgibacillus	0	1	0	0	1	0
5	0.2.10.1.2.5	Planococcaceae	1	1	0	0	1	0
6	0.2.10.1.2.5.1	Filibacter	0	1	0	0	1	0
5	0.2.10.1.2.6	Thermoactinomycetaceae	1	1	0	1	1	1
6	0.2.10.1.2.6.1	Desmospora	0	1	0	1	1	1
3	0.2.10.2	Clostridia	3	35	2	7	18	9
4	0.2.10.2.1	Thermoanaerobacterales	2	6	1	3	1	1
5	0.2.10.2.1.1	Thermoanaerobacteraceae	5	5	1	3	1	0
6	0.2.10.2.1.1.1	Ammonifex	0	1	0	1	0	0
6	0.2.10.2.1.1.2	Caldanaerovirga	0	1	0	1	0	0
6	0.2.10.2.1.1.3	Carboxydibrachium	0	1	0	1	0	0
6	0.2.10.2.1.1.4	Thermanaeromonas	0	1	0	0	1	0
6	0.2.10.2.1.1.5	Thermovenabulum	0	1	1	0	0	0
5	0.2.10.2.1.2	Thermodesulfobiaceae	1	1	0	0	0	1
6	0.2.10.2.1.2.1	Thermodesulfobium	0	1	0	0	0	1
4	0.2.10.2.2	Clostridiales	9	27	1	4	15	8

5	0.2.10.2.2.1	Lachnospiraceae	3	3	0	1	0	2
6	0.2.10.2.2.1.1	Johnsonella	0	1	0	1	0	0
6	0.2.10.2.2.1.2	Marvinbryantia	0	1	0	0	0	1
6	0.2.10.2.2.1.3	Syntrophococcus	0	1	0	0	0	1
5	0.2.10.2.2.2	Ruminococcaceae	5	5	0	1	3	1
6	0.2.10.2.2.2.1	Acetivibrio	0	1	0	0	1	0
6	0.2.10.2.2.2.2	Butyricicoccus	0	1	0	0	1	0
6	0.2.10.2.2.2.3	Fastidiosipila	0	1	0	0	0	1
6	0.2.10.2.2.2.4	Oscillibacter	0	1	0	0	1	0
6	0.2.10.2.2.2.5	Papillibacter	0	1	0	1	0	0
5	0.2.10.2.2.3	Clostridiaceae	5	5	0	1	3	1
6	0.2.10.2.2.3.1	Anoxynatronum	0	1	0	0	1	0
6	0.2.10.2.2.3.2	Caloranaerobacter	0	1	0	0	1	0
6	0.2.10.2.2.3.3	Clostridium	0	1	0	1	0	0
6	0.2.10.2.2.3.4	Geosporobacter	0	1	0	0	0	1
6	0.2.10.2.2.3.5	Natronincola	0	1	0	0	1	0
5	0.2.10.2.2.4	Incertae_Sedis_XI	4	4	0	0	4	0
6	0.2.10.2.2.4.1	Anaerococcus	0	1	0	0	1	0
6	0.2.10.2.2.4.2	Finegoldia	0	1	0	0	1	0
6	0.2.10.2.2.4.3	Parvimonas	0	1	0	0	1	0
6	0.2.10.2.2.4.4	Peptoniphilus	0	1	0	0	1	0
5	0.2.10.2.2.5	Incertae_Sedis_XIII	1	1	0	0	1	0
6	0.2.10.2.2.5.1	Anaerovorax	0	1	0	0	1	0
5	0.2.10.2.2.6	Incertae_Sedis_XIV	2	2	0	0	1	1
6	0.2.10.2.2.6.1	Dethiosulfatibacter	0	1	0	0	0	1
6	0.2.10.2.2.6.2	Proteocatella	0	1	0	0	1	0
5	0.2.10.2.2.7	Incertae_Sedis_XVIII	1	1	0	0	1	1
6	0.2.10.2.2.7.1	Symbiobacterium	0	1	0	0	1	1
5	0.2.10.2.2.8	Peptococcaceae	2	2	0	1	0	1
6	0.2.10.2.2.8.1	Cryptanaerobacter	0	1	0	1	0	0
6	0.2.10.2.2.8.2	Desulfurispora	0	1	0	0	0	1
5	0.2.10.2.2.9	Veillonellaceae	4	4	1	0	2	1
6	0.2.10.2.2.9.1	Anaeroarcus	0	1	1	0	0	0
6	0.2.10.2.2.9.2	Anaeromusa	0	1	0	0	1	0
6	0.2.10.2.2.9.3	Propionispira	0	1	0	0	0	1
6	0.2.10.2.2.9.4	Zymophilus	0	1	0	0	1	0
4	0.2.10.2.3	Natranaerobiales	1	2	0	0	2	0
5	0.2.10.2.3.1	Natranaerobiaceae	2	2	0	0	2	0
6	0.2.10.2.3.1.1	Natranaerobius	0	1	0	0	1	0
6	0.2.10.2.3.1.2	Natronovirga	0	1	0	0	1	0
3	0.2.10.3	Erysipelotrichi	1	2	0	2	0	2
4	0.2.10.3.1	Erysipelotrichales	1	2	0	2	0	2
5	0.2.10.3.1.1	Erysipelotrichaceae	2	2	0	2	0	2
6	0.2.10.3.1.1.1	Catenibacterium	0	1	0	1	0	1
6	0.2.10.3.1.1.2	Sharpea	0	1	0	1	0	1
2	0.2.11	Fusobacteria	1	2	0	0	1	1
3	0.2.11.1	Fusobacteria	1	2	0	0	1	1
4	0.2.11.1.1	Fusobacteriales	1	2	0	0	1	1
5	0.2.11.1.1.1	Leptotrichiaceae	2	2	0	0	1	1
6	0.2.11.1.1.1.1	Sebaldella	0	1	0	0	0	1
6	0.2.11.1.1.1.2	Streptobacillus	0	1	0	0	1	0
2	0.2.12	Gemmatimonadetes	1	1	0	0	1	0
3	0.2.12.1	Gemmatimonadetes	1	1	0	0	1	0
4	0.2.12.1.1	Gemmatimonadales	1	1	0	0	1	0
5	0.2.12.1.1.1	Gemmatimonadaceae	1	1	0	0	1	0
6	0.2.12.1.1.1.1	Gemmatimonas	0	1	0	0	1	0
2	0.2.13	Lentisphaerae	1	2	0	2	0	0
3	0.2.13.1	Lentisphaeria	2	2	0	2	0	0
4	0.2.13.1.1	Lentisphaerales	1	1	0	1	0	0
5	0.2.13.1.1.1	Lentisphaeraceae	1	1	0	1	0	0
6	0.2.13.1.1.1.1	Lentisphaera	0	1	0	1	0	0
4	0.2.13.1.2	Victivallales	1	1	0	1	0	0
5	0.2.13.1.2.1	Victivallaceae	1	1	0	1	0	0
6	0.2.13.1.2.1.1	Victivallis	0	1	0	1	0	0
2	0.2.14	Nitrospira	1	1	0	1	1	1
3	0.2.14.1	Nitrospira	1	1	0	1	1	1
4	0.2.14.1.1	Nitrospirales	1	1	0	1	1	1
5	0.2.14.1.1.1	Nitrospiraceae	1	1	0	1	1	1
6	0.2.14.1.1.1.1	Nitrospira	0	1	0	1	1	1
2	0.2.15	Planctomycetes	1	9	8	2	7	5
3	0.2.15.1	Planctomycetacia	1	9	8	2	7	5
4	0.2.15.1.1	Planctomycetales	1	9	8	2	7	5
5	0.2.15.1.1.1	Planctomycetaceae	9	9	8	2	7	5
6	0.2.15.1.1.1.1	Blastopirellula	0	1	1	0	1	1
6	0.2.15.1.1.1.2	Gemmata	0	1	1	0	1	0
6	0.2.15.1.1.1.3	Isosphaera	0	1	0	0	1	1
6	0.2.15.1.1.1.4	Pirellula	0	1	1	1	1	1
6	0.2.15.1.1.1.5	Planctomyces	0	1	1	0	0	0
6	0.2.15.1.1.1.6	Rhodopirellula	0	1	1	0	1	0
6	0.2.15.1.1.1.7	Schlesneria	0	1	1	1	1	0
6	0.2.15.1.1.1.8	Singulisphaera	0	1	1	0	0	1
6	0.2.15.1.1.1.9	Zavarzinella	0	1	1	0	1	1
2	0.2.16	Proteobacteria	5	363	60	15	341	28
3	0.2.16.1	Alphaproteobacteria	11	163	32	6	158	7
4	0.2.16.1.1	Parvularculales	1	1	1	0	1	0
5	0.2.16.1.1.1	Parvularculaceae	1	1	1	0	1	0
6	0.2.16.1.1.1.1	Parvularcula	0	1	1	0	1	0

4	0.2.16.1.2	Alphaproteobacteria_order_inc	1	4	1	0	4	0
5	0.2.16.1.2.1	Alphaproteobacteria_incertae_s	4	4	1	0	4	0
6	0.2.16.1.2.1.1	Elioraea	0	1	0	0	1	0
6	0.2.16.1.2.1.2	Geminicoccus	0	1	1	0	1	0
6	0.2.16.1.2.1.3	Insolitispirillum	0	1	0	0	1	0
6	0.2.16.1.2.1.4	Novispirillum	0	1	0	0	1	0
4	0.2.16.1.3	Caulobacterales	2	11	4	0	10	0
5	0.2.16.1.3.1	Caulobacteraceae	4	4	0	0	4	0
6	0.2.16.1.3.1.1	Asticcacaulis	0	1	0	0	1	0
6	0.2.16.1.3.1.2	Brevundimonas	0	1	0	0	1	0
6	0.2.16.1.3.1.3	Caulobacter	0	1	0	0	1	0
6	0.2.16.1.3.1.4	Phenylobacterium	0	1	0	0	1	0
5	0.2.16.1.3.2	Hyphomonadaceae	7	7	4	0	6	0
6	0.2.16.1.3.2.1	Hellea	0	1	1	0	1	0
6	0.2.16.1.3.2.2	Hirschia	0	1	1	0	1	0
6	0.2.16.1.3.2.3	Hyphomonas	0	1	0	0	1	0
6	0.2.16.1.3.2.4	Oceanicaulis	0	1	1	0	1	0
6	0.2.16.1.3.2.5	Ponticaulis	0	1	0	0	1	0
6	0.2.16.1.3.2.6	Robiginitomaculum	0	1	0	0	1	0
6	0.2.16.1.3.2.7	Woodsholea	0	1	1	0	0	0
4	0.2.16.1.4	Kiloniellales	1	1	1	0	1	0
5	0.2.16.1.4.1	Kiloniellaceae	1	1	1	0	1	0
6	0.2.16.1.4.1.1	Kiloniella	0	1	1	0	1	0
4	0.2.16.1.5	Kordiimonadales	1	1	0	0	1	0
5	0.2.16.1.5.1	Kordiimonadaceae	1	1	0	0	1	0
6	0.2.16.1.5.1.1	Kordiimonas	0	1	0	0	1	0
4	0.2.16.1.6	Rhizobiales	14	66	18	1	65	3
5	0.2.16.1.6.1	Aurantimonadaceae	3	3	1	0	3	1
6	0.2.16.1.6.1.1	Aurantimonas	0	1	0	0	1	0
6	0.2.16.1.6.1.2	Fulvimarina	0	1	0	0	1	0
6	0.2.16.1.6.1.3	Martelella	0	1	1	0	1	1
5	0.2.16.1.6.2	Bartonellaceae	1	1	0	0	1	0
6	0.2.16.1.6.2.1	Bartonella	0	1	0	0	1	0
5	0.2.16.1.6.3	Beijerinckiaceae	5	5	4	1	5	0
6	0.2.16.1.6.3.1	Beijerinckia	0	1	1	0	1	0
6	0.2.16.1.6.3.2	Chelatococcus	0	1	1	0	1	0
6	0.2.16.1.6.3.3	Methylocapsa	0	1	1	0	1	0
6	0.2.16.1.6.3.4	Methylocella	0	1	1	0	1	0
6	0.2.16.1.6.3.5	Methylovirgula	0	1	0	1	1	0
5	0.2.16.1.6.4	Bradyrhizobiaceae	8	8	2	0	8	0
6	0.2.16.1.6.4.1	Afipia	0	1	0	0	1	0
6	0.2.16.1.6.4.2	Agromonas	0	1	0	0	1	0
6	0.2.16.1.6.4.3	Balneimonas	0	1	0	0	1	0
6	0.2.16.1.6.4.4	Bosea	0	1	1	0	1	0
6	0.2.16.1.6.4.5	Bradyrhizobium	0	1	0	0	1	0
6	0.2.16.1.6.4.6	Nitrobacter	0	1	0	0	1	0
6	0.2.16.1.6.4.7	Rhodoblastus	0	1	0	0	1	0
6	0.2.16.1.6.4.8	Rhodopseudomonas	0	1	1	0	1	0
5	0.2.16.1.6.5	Brucellaceae	5	5	0	0	5	1
6	0.2.16.1.6.5.1	Brucella	0	1	0	0	1	0
6	0.2.16.1.6.5.2	Daeguia	0	1	0	0	1	1
6	0.2.16.1.6.5.3	Mycoplana	0	1	0	0	1	0
6	0.2.16.1.6.5.4	Ochrobactrum	0	1	0	0	1	0
6	0.2.16.1.6.5.5	Pseudochrobactrum	0	1	0	0	1	0
5	0.2.16.1.6.6	Cohaesibacteraceae	1	1	0	0	1	0
6	0.2.16.1.6.6.1	Cohaesibacter	0	1	0	0	1	0
5	0.2.16.1.6.7	Hyphomicrobiaceae	12	12	7	0	11	0
6	0.2.16.1.6.7.1	Angulomicrobium	0	1	0	0	1	0
6	0.2.16.1.6.7.2	Blastochloris	0	1	1	0	1	0
6	0.2.16.1.6.7.3	Cucumibacter	0	1	0	0	1	0
6	0.2.16.1.6.7.4	Devosia	0	1	1	0	1	0
6	0.2.16.1.6.7.5	Filomicrobium	0	1	0	0	1	0
6	0.2.16.1.6.7.6	Hyphomicrobium	0	1	1	0	1	0
6	0.2.16.1.6.7.7	Maritalea	0	1	0	0	1	0
6	0.2.16.1.6.7.8	Methylorhabdus	0	1	1	0	0	0
6	0.2.16.1.6.7.9	Pedomicrobium	0	1	1	0	1	0
6	0.2.16.1.6.7.1	Prosthecomicrobium	0	1	1	0	1	0
6	0.2.16.1.6.7.1	Rhodoplanes	0	1	1	0	1	0
6	0.2.16.1.6.7.1	Zhangella	0	1	0	0	1	0
5	0.2.16.1.6.8	Methylobacteriaceae	3	3	0	0	3	0
6	0.2.16.1.6.8.1	Meganema	0	1	0	0	1	0
6	0.2.16.1.6.8.2	Methylobacterium	0	1	0	0	1	0
6	0.2.16.1.6.8.3	Microvirga	0	1	0	0	1	0
5	0.2.16.1.6.9	Methylocystaceae	5	5	1	0	5	1
6	0.2.16.1.6.9.1	Methylocystis	0	1	0	0	1	0
6	0.2.16.1.6.9.2	Methylopila	0	1	1	0	1	1
6	0.2.16.1.6.9.3	Methylosinus	0	1	0	0	1	0
6	0.2.16.1.6.9.4	Pleomorphomonas	0	1	0	0	1	0
6	0.2.16.1.6.9.5	Terasakiella	0	1	0	0	1	0
5	0.2.16.1.6.10	Phyllobacteriaceae	6	6	2	0	6	0
6	0.2.16.1.6.10	Aminobacter	0	1	0	0	1	0
6	0.2.16.1.6.10	Hoeflea	0	1	1	0	1	0
6	0.2.16.1.6.10	Mesorhizobium	0	1	1	0	1	0
6	0.2.16.1.6.10	Nitratireductor	0	1	0	0	1	0
6	0.2.16.1.6.10	Phyllobacterium	0	1	0	0	1	0
6	0.2.16.1.6.10	Pseudaminobacter	0	1	0	0	1	0
5	0.2.16.1.6.11	Rhizobiaceae	4	4	0	0	4	0

6	0.2.16.1.6.11	Ensifer	0	1	0	0	1	0
6	0.2.16.1.6.11	Kaistia	0	1	0	0	1	0
6	0.2.16.1.6.11	Rhizobium	0	1	0	0	1	0
6	0.2.16.1.6.11	Sinorhizobium	0	1	0	0	1	0
5	0.2.16.1.6.12	Rhizobiales_incertae_sedis	1	1	0	0	1	0
6	0.2.16.1.6.12	Amorphus	0	1	0	0	1	0
5	0.2.16.1.6.13	Rhodobiaceae	5	5	0	0	5	0
6	0.2.16.1.6.13	Afifella	0	1	0	0	1	0
6	0.2.16.1.6.13	Anderseniella	0	1	0	0	1	0
6	0.2.16.1.6.13	Parvibaculum	0	1	0	0	1	0
6	0.2.16.1.6.13	Rhodobium	0	1	0	0	1	0
6	0.2.16.1.6.13	Roseospirillum	0	1	0	0	1	0
5	0.2.16.1.6.14	Xanthobacteraceae	7	7	1	0	7	0
6	0.2.16.1.6.14	Ancylobacter	0	1	0	0	1	0
6	0.2.16.1.6.14	Azorhizobium	0	1	0	0	1	0
6	0.2.16.1.6.14	Labrys	0	1	0	0	1	0
6	0.2.16.1.6.14	Pseudolabrys	0	1	0	0	1	0
6	0.2.16.1.6.14	Pseudoxanthobacter	0	1	0	0	1	0
6	0.2.16.1.6.14	Starkeya	0	1	0	0	1	0
6	0.2.16.1.6.14	Xanthobacter	0	1	1	0	1	0
4	0.2.16.1.7	Rhodobacterales	1	25	2	1	23	2
5	0.2.16.1.7.1	Rhodobacteraceae	25	25	2	1	23	2
6	0.2.16.1.7.1.1	Ahrensia	0	1	0	0	1	0
6	0.2.16.1.7.1.2	Albidovulum	0	1	0	0	1	0
6	0.2.16.1.7.1.3	Albimonas	0	1	0	0	1	0
6	0.2.16.1.7.1.4	Amaricoccus	0	1	0	0	1	0
6	0.2.16.1.7.1.5	Antarctobacter	0	1	0	0	0	1
6	0.2.16.1.7.1.6	Catellibacterium	0	1	0	0	1	0
6	0.2.16.1.7.1.7	Haematobacter	0	1	0	0	1	0
6	0.2.16.1.7.1.8	Jannaschia	0	1	0	0	1	0
6	0.2.16.1.7.1.9	Ketogulonicigenium	0	1	0	0	1	0
6	0.2.16.1.7.1.1	Labrenzia	0	1	0	0	1	0
6	0.2.16.1.7.1.1	Loktanella	0	1	0	0	1	0
6	0.2.16.1.7.1.1	Lutimaribacter	0	1	0	1	1	0
6	0.2.16.1.7.1.1	Maritimibacter	0	1	0	0	1	0
6	0.2.16.1.7.1.1	Nesiotobacter	0	1	0	0	1	0
6	0.2.16.1.7.1.1	Palleronia	0	1	0	0	1	0
6	0.2.16.1.7.1.1	Pannonibacter	0	1	1	0	1	0
6	0.2.16.1.7.1.1	Pseudovibrio	0	1	0	0	1	0
6	0.2.16.1.7.1.1	Rhodobaca	0	1	0	0	1	0
6	0.2.16.1.7.1.1	Rhodobacter	0	1	1	0	1	0
6	0.2.16.1.7.1.2	Rhodothalassium	0	1	0	0	1	0
6	0.2.16.1.7.1.2	Rhodovulum	0	1	0	0	1	0
6	0.2.16.1.7.1.2	Roseinatronobacter	0	1	0	0	1	0
6	0.2.16.1.7.1.2	Rubellimicrobium	0	1	0	0	1	0
6	0.2.16.1.7.1.2	Salinihabitans	0	1	0	0	1	0
6	0.2.16.1.7.1.2	Tropicibacter	0	1	0	0	0	1
4	0.2.16.1.8	Rhodospirillales	2	40	4	4	39	2
5	0.2.16.1.8.1	Acetobacteraceae	19	19	0	3	18	2
6	0.2.16.1.8.1.1	Acidicaldus	0	1	0	1	1	0
6	0.2.16.1.8.1.2	Acidiphilium	0	1	0	0	1	0
6	0.2.16.1.8.1.3	Acidisoma	0	1	0	0	1	0
6	0.2.16.1.8.1.4	Acidisphaera	0	1	0	0	1	0
6	0.2.16.1.8.1.5	Acidocella	0	1	0	0	1	0
6	0.2.16.1.8.1.6	Acidomonas	0	1	0	0	0	1
6	0.2.16.1.8.1.7	Asaia	0	1	0	0	1	0
6	0.2.16.1.8.1.8	Belnapia	0	1	0	0	1	1
6	0.2.16.1.8.1.9	Craurococcus	0	1	0	0	1	0
6	0.2.16.1.8.1.1	Gluconacetobacter	0	1	0	0	1	0
6	0.2.16.1.8.1.1	Granulibacter	0	1	0	0	1	0
6	0.2.16.1.8.1.1	Kozakia	0	1	0	0	1	0
6	0.2.16.1.8.1.1	Neoasaia	0	1	0	0	1	0
6	0.2.16.1.8.1.1	Paracraurococcus	0	1	0	0	1	0
6	0.2.16.1.8.1.1	Rhodopila	0	1	0	1	1	0
6	0.2.16.1.8.1.1	Roseomonas	0	1	0	0	1	0
6	0.2.16.1.8.1.1	Rubritepida	0	1	0	0	1	0
6	0.2.16.1.8.1.1	Saccharibacter	0	1	0	0	1	0
6	0.2.16.1.8.1.1	Stella	0	1	0	1	1	0
5	0.2.16.1.8.2	Rhodospirillaceae	21	21	4	1	21	0
6	0.2.16.1.8.2.1	Azospirillum	0	1	1	0	1	0
6	0.2.16.1.8.2.2	Caenispirillum	0	1	0	0	1	0
6	0.2.16.1.8.2.3	Defluviicoccus	0	1	0	0	1	0
6	0.2.16.1.8.2.4	Fodinicurvata	0	1	1	0	1	0
6	0.2.16.1.8.2.5	Inquilinus	0	1	1	1	1	0
6	0.2.16.1.8.2.6	Magnetospirillum	0	1	0	0	1	0
6	0.2.16.1.8.2.7	Marispirillum	0	1	0	0	1	0
6	0.2.16.1.8.2.8	Nisaea	0	1	0	0	1	0
6	0.2.16.1.8.2.9	Oceanibaculum	0	1	1	0	1	0
6	0.2.16.1.8.2.1	Pelagibius	0	1	0	0	1	0
6	0.2.16.1.8.2.1	Phaeospirillum	0	1	0	0	1	0
6	0.2.16.1.8.2.1	Rhodocista	0	1	0	0	1	0
6	0.2.16.1.8.2.1	Rhodospira	0	1	0	0	1	0
6	0.2.16.1.8.2.1	Rhodospirillum	0	1	0	0	1	0
6	0.2.16.1.8.2.1	Rhodovibrio	0	1	0	0	1	0
6	0.2.16.1.8.2.1	Roseospira	0	1	0	0	1	0
6	0.2.16.1.8.2.1	Skermanella	0	1	0	0	1	0
6	0.2.16.1.8.2.1	Telmatospirillum	0	1	0	0	1	0

6	0.2.16.1.8.2.1	Thalassobaculum	0	1	0	0	1	0
6	0.2.16.1.8.2.2	Thalassospira	0	1	0	0	1	0
6	0.2.16.1.8.2.2	Tistrella	0	1	0	0	1	0
4	0.2.16.1.9	Rickettsiales	2	3	0	0	3	0
5	0.2.16.1.9.1	Anaplasmataceae	1	1	0	0	1	0
6	0.2.16.1.9.1.1	Ehrlichia	0	1	0	0	1	0
5	0.2.16.1.9.2	Rickettsiaceae	2	2	0	0	2	0
6	0.2.16.1.9.2.1	Orientia	0	1	0	0	1	0
6	0.2.16.1.9.2.2	Rickettsia	0	1	0	0	1	0
4	0.2.16.1.10	Sneathiellales	1	1	0	0	1	0
5	0.2.16.1.10.1	Sneathiellaceae	1	1	0	0	1	0
6	0.2.16.1.10.1	Sneathiella	0	1	0	0	1	0
4	0.2.16.1.11	Sphingomonadales	2	10	1	0	10	0
5	0.2.16.1.11.1	Erythrobacteraceae	2	2	0	0	2	0
6	0.2.16.1.11.1.	Croceicoccus	0	1	0	0	1	0
6	0.2.16.1.11.1.	Erythromicrobium	0	1	0	0	1	0
5	0.2.16.1.11.2	Sphingomonadaceae	8	8	1	0	8	0
6	0.2.16.1.11.2.	Blastomonas	0	1	0	0	1	0
6	0.2.16.1.11.2.	Novosphingobium	0	1	0	0	1	0
6	0.2.16.1.11.2.	Sandaracinobacter	0	1	0	0	1	0
6	0.2.16.1.11.2.	Sandarakinorhabdus	0	1	0	0	1	0
6	0.2.16.1.11.2.	Sphingobium	0	1	0	0	1	0
6	0.2.16.1.11.2.	Sphingomonas	0	1	1	0	1	0
6	0.2.16.1.11.2.	Sphingopyxis	0	1	0	0	1	0
6	0.2.16.1.11.2.	Sphingosinicella	0	1	0	0	1	0
3	0.2.16.2	Betaproteobacteria	4	31	6	2	25	4
4	0.2.16.2.1	Burkholderiales	5	17	5	1	13	2
5	0.2.16.2.1.1	Alcaligenaceae	3	3	1	0	2	0
6	0.2.16.2.1.1.1	Castellaniella	0	1	0	0	1	0
6	0.2.16.2.1.1.2	Derxia	0	1	1	0	0	0
6	0.2.16.2.1.1.3	Parasutterella	0	1	0	0	1	0
5	0.2.16.2.1.2	Burkholderiaceae	3	3	0	0	3	1
6	0.2.16.2.1.2.1	Burkholderia	0	1	0	0	1	0
6	0.2.16.2.1.2.2	Chitinimonas	0	1	0	0	1	0
6	0.2.16.2.1.2.3	Wautersia	0	1	0	0	1	1
5	0.2.16.2.1.3	Burkholderiales_incertae_sedis	4	4	1	1	4	0
6	0.2.16.2.1.3.1	Aquabacterium	0	1	0	0	1	0
6	0.2.16.2.1.3.2	Ideonella	0	1	0	0	1	0
6	0.2.16.2.1.3.3	Rhizobacter	0	1	0	0	1	0
6	0.2.16.2.1.3.4	Thiobacter	0	1	1	1	1	0
5	0.2.16.2.1.4	Comamonadaceae	5	5	1	0	4	1
6	0.2.16.2.1.4.1	Acidovorax	0	1	0	0	1	0
6	0.2.16.2.1.4.2	Delftia	0	1	0	0	1	0
6	0.2.16.2.1.4.3	Polaromonas	0	1	0	0	1	0
6	0.2.16.2.1.4.4	Tepidicella	0	1	0	0	0	1
6	0.2.16.2.1.4.5	Variovorax	0	1	1	0	1	0
5	0.2.16.2.1.5	Oxalobacteraceae	2	2	2	0	0	0
6	0.2.16.2.1.5.1	Herbaspirillum	0	1	1	0	0	0
6	0.2.16.2.1.5.2	Massilia	0	1	1	0	0	0
4	0.2.16.2.2	Hydrogenophilales	1	2	0	1	2	1
5	0.2.16.2.2.1	Hydrogenophilaceae	2	2	0	1	2	1
6	0.2.16.2.2.1.1	Hydrogenophilus	0	1	0	1	1	0
6	0.2.16.2.2.1.2	Tepidiphilus	0	1	0	0	1	1
4	0.2.16.2.3	Neisseriales	1	7	0	0	7	0
5	0.2.16.2.3.1	Neisseriaceae	7	7	0	0	7	0
6	0.2.16.2.3.1.1	Andreprevotia	0	1	0	0	1	0
6	0.2.16.2.3.1.2	Aquitalea	0	1	0	0	1	0
6	0.2.16.2.3.1.3	Chitinilyticum	0	1	0	0	1	0
6	0.2.16.2.3.1.4	Deefgea	0	1	0	0	1	0
6	0.2.16.2.3.1.5	Paludibacterium	0	1	0	0	1	0
6	0.2.16.2.3.1.6	Silvimonas	0	1	0	0	1	0
6	0.2.16.2.3.1.7	Vitreoscilla	0	1	0	0	1	0
4	0.2.16.2.4	Rhodocyclales	1	5	1	0	3	1
5	0.2.16.2.4.1	Rhodocyclaceae	5	5	1	0	3	1
6	0.2.16.2.4.1.1	Azonexus	0	1	0	0	1	0
6	0.2.16.2.4.1.2	Dechloromonas	0	1	0	0	1	0
6	0.2.16.2.4.1.3	Ferribacterium	0	1	0	0	1	0
6	0.2.16.2.4.1.4	Methyloversatilis	0	1	1	0	0	0
6	0.2.16.2.4.1.5	Quatrionicoccus	0	1	0	0	0	1
3	0.2.16.3	Deltaproteobacteria	8	49	15	1	45	10
4	0.2.16.3.1	Bdellovibrionales	2	2	0	0	2	0
5	0.2.16.3.1.1	Bacteriovoracaceae	1	1	0	0	1	0
6	0.2.16.3.1.1.1	Peredibacter	0	1	0	0	1	0
5	0.2.16.3.1.2	Bdellovibrionaceae	1	1	0	0	1	0
6	0.2.16.3.1.2.1	Bdellovibrio	0	1	0	0	1	0
4	0.2.16.3.2	Desulfarculales	1	1	1	0	1	0
5	0.2.16.3.2.1	Desulfarculaceae	1	1	1	0	1	0
6	0.2.16.3.2.1.1	Desulfarculus	0	1	1	0	1	0
4	0.2.16.3.3	Desulfobacterales	2	14	1	0	14	2
5	0.2.16.3.3.1	Desulfobacteraceae	9	9	1	0	9	1
6	0.2.16.3.3.1.1	Desulfatibacillum	0	1	0	0	1	0
6	0.2.16.3.3.1.2	Desulfatiferula	0	1	0	0	1	0
6	0.2.16.3.3.1.3	Desulfatirhabdium	0	1	0	0	1	0
6	0.2.16.3.3.1.4	Desulfobacula	0	1	0	0	1	0
6	0.2.16.3.3.1.5	Desulfocella	0	1	0	0	1	1
6	0.2.16.3.3.1.6	Desulfococcus	0	1	0	0	1	0
6	0.2.16.3.3.1.7	Desulfoluna	0	1	0	0	1	0

6	0.2.16.3.3.1.8	Desulforegula	0	1	1	0	1	0
6	0.2.16.3.3.1.9	Desulfospira	0	1	0	0	1	0
5	0.2.16.3.3.2	Desulfobulbaceae	5	5	0	0	5	1
6	0.2.16.3.3.2.1	Desulfocapsa	0	1	0	0	1	0
6	0.2.16.3.3.2.2	Desulfopila	0	1	0	0	1	1
6	0.2.16.3.3.2.3	Desulforhopalus	0	1	0	0	1	0
6	0.2.16.3.3.2.4	Desulfotalea	0	1	0	0	1	0
6	0.2.16.3.3.2.5	Desulfurivibrio	0	1	0	0	1	0
4	0.2.16.3.4	Desulfovibrionales	2	5	2	0	2	2
5	0.2.16.3.4.1	Desulfohalobiaceae	2	2	1	0	1	0
6	0.2.16.3.4.1.1	Desulfonatronospira	0	1	0	0	1	0
6	0.2.16.3.4.1.2	Desulfovermiculus	0	1	1	0	0	0
5	0.2.16.3.4.2	Desulfovibrionaceae	3	3	1	0	1	2
6	0.2.16.3.4.2.1	Bilophila	0	1	0	0	0	1
6	0.2.16.3.4.2.2	Desulfocurvus	0	1	1	0	1	0
6	0.2.16.3.4.2.3	Lawsonia	0	1	0	0	0	1
4	0.2.16.3.5	Desulfurellales	1	1	0	0	1	0
5	0.2.16.3.5.1	Desulfurellaceae	1	1	0	0	1	0
6	0.2.16.3.5.1.1	Hippea	0	1	0	0	1	0
4	0.2.16.3.6	Desulfuromonadales	2	3	2	0	3	0
5	0.2.16.3.6.1	Desulfuromonadaceae	1	1	0	0	1	0
6	0.2.16.3.6.1.1	Malonomonas	0	1	0	0	1	0
5	0.2.16.3.6.2	Geobacteraceae	2	2	2	0	2	0
6	0.2.16.3.6.2.1	Geopsychrobacter	0	1	1	0	1	0
6	0.2.16.3.6.2.2	Geothermobacter	0	1	1	0	1	0
4	0.2.16.3.7	Myxococcales	7	15	8	1	14	5
5	0.2.16.3.7.1	Haliangiaceae	1	1	0	0	1	0
6	0.2.16.3.7.1.1	Haliangium	0	1	0	0	1	0
5	0.2.16.3.7.2	Cystobacteraceae	4	4	1	0	3	2
6	0.2.16.3.7.2.1	Anaeromyxobacter	0	1	1	0	1	1
6	0.2.16.3.7.2.2	Archangium	0	1	0	0	0	1
6	0.2.16.3.7.2.3	Hyalangium	0	1	0	0	1	0
6	0.2.16.3.7.2.4	Melittangium	0	1	0	0	1	0
5	0.2.16.3.7.3	Kofleriaceae	1	1	1	0	1	0
6	0.2.16.3.7.3.1	Kofleria	0	1	1	0	1	0
5	0.2.16.3.7.4	Myxococcaceae	2	2	2	0	2	0
6	0.2.16.3.7.4.1	Corallococcus	0	1	1	0	1	0
6	0.2.16.3.7.4.2	Pyxidicoccus	0	1	1	0	1	0
5	0.2.16.3.7.5	Nannocystaceae	3	3	1	1	3	1
6	0.2.16.3.7.5.1	Enhygromyxa	0	1	0	0	1	0
6	0.2.16.3.7.5.2	Nannocystis	0	1	1	1	1	0
6	0.2.16.3.7.5.3	Plesiocystis	0	1	0	0	1	1
5	0.2.16.3.7.6	Phaselicystidaceae	1	1	1	0	1	0
6	0.2.16.3.7.6.1	Phaselicystis	0	1	1	0	1	0
5	0.2.16.3.7.7	Polyangiaceae	3	3	2	0	3	2
6	0.2.16.3.7.7.1	Byssovorax	0	1	1	0	1	0
6	0.2.16.3.7.7.2	Chondromyces	0	1	0	0	1	1
6	0.2.16.3.7.7.3	Sorangium	0	1	1	0	1	1
4	0.2.16.3.8	Syntrophobacterales	2	8	1	0	8	1
5	0.2.16.3.8.1	Syntrophaceae	4	4	0	0	4	0
6	0.2.16.3.8.1.1	Desulfobacca	0	1	0	0	1	0
6	0.2.16.3.8.1.2	Desulfomonile	0	1	0	0	1	0
6	0.2.16.3.8.1.3	Smithella	0	1	0	0	1	0
6	0.2.16.3.8.1.4	Syntrophus	0	1	0	0	1	0
5	0.2.16.3.8.2	Syntrophobacteraceae	4	4	1	0	4	1
6	0.2.16.3.8.2.1	Desulfacinum	0	1	0	0	1	0
6	0.2.16.3.8.2.2	Desulfoglaeba	0	1	1	0	1	0
6	0.2.16.3.8.2.3	Desulfovirga	0	1	0	0	1	0
6	0.2.16.3.8.2.4	Thermodesulforhabdus	0	1	0	0	1	1
3	0.2.16.4	Epsilonproteobacteria	2	5	0	0	5	1
4	0.2.16.4.1	Campylobacterales	2	2	0	0	2	0
5	0.2.16.4.1.1	Hydrogenimonaceae	1	1	0	0	1	0
6	0.2.16.4.1.1.1	Hydrogenimonas	0	1	0	0	1	0
5	0.2.16.4.1.2	Helicobacteraceae	1	1	0	0	1	0
6	0.2.16.4.1.2.1	Sulfuricurvum	0	1	0	0	1	0
4	0.2.16.4.2	Nautiliales	1	3	0	0	3	1
5	0.2.16.4.2.1	Nautiliaceae	3	3	0	0	3	1
6	0.2.16.4.2.1.1	Lebetimonas	0	1	0	0	1	0
6	0.2.16.4.2.1.2	Nitratifractor	0	1	0	0	1	0
6	0.2.16.4.2.1.3	Thioreductor	0	1	0	0	1	1
3	0.2.16.5	Gammaproteobacteria	16	115	7	6	108	6
4	0.2.16.5.1	Enterobacteriales	1	14	1	4	13	0
5	0.2.16.5.1.1	Enterobacteriaceae	14	14	1	4	13	0
6	0.2.16.5.1.1.1	Buttiauxella	0	1	0	0	1	0
6	0.2.16.5.1.1.2	Cedecea	0	1	0	0	1	0
6	0.2.16.5.1.1.3	Citrobacter	0	1	0	0	1	0
6	0.2.16.5.1.1.4	Enterobacter	0	1	0	1	1	0
6	0.2.16.5.1.1.5	Escherichia/Shigella	0	1	1	0	1	0
6	0.2.16.5.1.1.6	Klebsiella	0	1	0	1	0	0
6	0.2.16.5.1.1.7	Kluyvera	0	1	0	1	1	0
6	0.2.16.5.1.1.8	Morganella	0	1	0	0	1	0
6	0.2.16.5.1.1.9	Photorhabdus	0	1	0	0	1	0
6	0.2.16.5.1.1.1	Plesiomonas	0	1	0	0	1	0
6	0.2.16.5.1.1.1	Providencia	0	1	0	0	1	0
6	0.2.16.5.1.1.1	Rahnella	0	1	0	0	1	0
6	0.2.16.5.1.1.1	Raoultella	0	1	0	1	1	0
6	0.2.16.5.1.1.1	Serratia	0	1	0	0	1	0

4	0.2.16.5.2	Salinisphaerales	1	1	0	0	1	0
5	0.2.16.5.2.1	Salinisphaeraceae	1	1	0	0	1	0
6	0.2.16.5.2.1.1	Salinisphaera	0	1	0	0	1	0
4	0.2.16.5.3	Vibrionales	1	1	0	0	1	0
5	0.2.16.5.3.1	Vibrionaceae	1	1	0	0	1	0
6	0.2.16.5.3.1.1	Aliivibrio	0	1	0	0	1	0
4	0.2.16.5.4	Acidithiobacillales	1	1	0	0	1	0
5	0.2.16.5.4.1	Thermithiobacillaceae	1	1	0	0	1	0
6	0.2.16.5.4.1.1	Thermithiobacillus	0	1	0	0	1	0
4	0.2.16.5.5	Aeromonadales	1	2	0	0	2	0
5	0.2.16.5.5.1	Succinivibrionaceae	2	2	0	0	2	0
6	0.2.16.5.5.1.1	Ruminobacter	0	1	0	0	1	0
6	0.2.16.5.5.1.2	Succinivibrio	0	1	0	0	1	0
4	0.2.16.5.6	Alteromonadales	5	10	0	0	10	0
5	0.2.16.5.6.1	Alteromonadaceae	6	6	0	0	6	0
6	0.2.16.5.6.1.1	Aestuariibacter	0	1	0	0	1	0
6	0.2.16.5.6.1.2	Agarivorans	0	1	0	0	1	0
6	0.2.16.5.6.1.3	Bowmanella	0	1	0	0	1	0
6	0.2.16.5.6.1.4	Haliea	0	1	0	0	1	0
6	0.2.16.5.6.1.5	Marinimicrobium	0	1	0	0	1	0
6	0.2.16.5.6.1.6	Salinimonas	0	1	0	0	1	0
5	0.2.16.5.6.2	Ferrimonadaceae	1	1	0	0	1	0
6	0.2.16.5.6.2.1	Paraferrimonas	0	1	0	0	1	0
5	0.2.16.5.6.3	Moritellaceae	1	1	0	0	1	0
6	0.2.16.5.6.3.1	Paramoritella	0	1	0	0	1	0
5	0.2.16.5.6.4	Pseudoalteromonadaceae	1	1	0	0	1	0
6	0.2.16.5.6.4.1	Pseudoalteromonas	0	1	0	0	1	0
5	0.2.16.5.6.5	Shewanellaceae	1	1	0	0	1	0
6	0.2.16.5.6.5.1	Shewanella	0	1	0	0	1	0
4	0.2.16.5.7	Cardiobacteriales	1	2	0	0	2	0
5	0.2.16.5.7.1	Cardiobacteriaceae	2	2	0	0	2	0
6	0.2.16.5.7.1.1	Cardiobacterium	0	1	0	0	1	0
6	0.2.16.5.7.1.2	Suttonella	0	1	0	0	1	0
4	0.2.16.5.8	Chromatiales	4	19	1	0	17	2
5	0.2.16.5.8.1	Chromatiaceae	11	11	1	0	10	0
6	0.2.16.5.8.1.1	Chromatium	0	1	0	0	1	0
6	0.2.16.5.8.1.2	Lamprocystis	0	1	0	0	1	0
6	0.2.16.5.8.1.3	Marichromatium	0	1	0	0	1	0
6	0.2.16.5.8.1.4	Nitrosococcus	0	1	1	0	0	0
6	0.2.16.5.8.1.5	Rhabdochromatium	0	1	0	0	1	0
6	0.2.16.5.8.1.6	Thiobaca	0	1	0	0	1	0
6	0.2.16.5.8.1.7	Thiococcus	0	1	0	0	1	0
6	0.2.16.5.8.1.8	Thiocystis	0	1	0	0	1	0
6	0.2.16.5.8.1.9	Thioflavicoccus	0	1	0	0	1	0
6	0.2.16.5.8.1.1	Thiohalocapsa	0	1	0	0	1	0
6	0.2.16.5.8.1.1	Thiophaeococcus	0	1	0	0	1	0
5	0.2.16.5.8.2	Ectothiorhodospiraceae	5	5	0	0	4	1
6	0.2.16.5.8.2.1	Alkalispirillum	0	1	0	0	0	1
6	0.2.16.5.8.2.2	Arhodomonas	0	1	0	0	1	0
6	0.2.16.5.8.2.3	Ectothiorhodosinus	0	1	0	0	1	0
6	0.2.16.5.8.2.4	Thioalkalispira	0	1	0	0	1	0
6	0.2.16.5.8.2.5	Thiorhodospira	0	1	0	0	1	0
5	0.2.16.5.8.3	Granulosicoccaceae	1	1	0	0	1	1
6	0.2.16.5.8.3.1	Granulosicoccus	0	1	0	0	1	1
5	0.2.16.5.8.4	Halothiobacillaceae	2	2	0	0	2	0
6	0.2.16.5.8.4.1	Halothiobacillus	0	1	0	0	1	0
6	0.2.16.5.8.4.2	Thiofaba	0	1	0	0	1	0
4	0.2.16.5.9	Gammaproteobacteria_order_ir	1	5	0	0	5	0
5	0.2.16.5.9.1	Gammaproteobacteria_incertae	5	5	0	0	5	0
6	0.2.16.5.9.1.1	Gilvimarinus	0	1	0	0	1	0
6	0.2.16.5.9.1.2	Methylohalomonas	0	1	0	0	1	0
6	0.2.16.5.9.1.3	Thiohalomonas	0	1	0	0	1	0
6	0.2.16.5.9.1.4	Thiohalophilus	0	1	0	0	1	0
6	0.2.16.5.9.1.5	Thiohalorhabdus	0	1	0	0	1	0
4	0.2.16.5.10	Legionellales	2	4	0	0	4	0
5	0.2.16.5.10.1	Coxiellaceae	1	1	0	0	1	0
6	0.2.16.5.10.1	Aquicella	0	1	0	0	1	0
5	0.2.16.5.10.2	Legionellaceae	3	3	0	0	3	0
6	0.2.16.5.10.2.	Fluoribacter	0	1	0	0	1	0
6	0.2.16.5.10.2.	Legionella	0	1	0	0	1	0
6	0.2.16.5.10.2.	Tatlockia	0	1	0	0	1	0
4	0.2.16.5.11	Methylococcales	1	6	0	0	6	0
5	0.2.16.5.11.1	Methylococcaceae	6	6	0	0	6	0
6	0.2.16.5.11.1.	Methylobacter	0	1	0	0	1	0
6	0.2.16.5.11.1.	Methylococcus	0	1	0	0	1	0
6	0.2.16.5.11.1.	Methylohalobius	0	1	0	0	1	0
6	0.2.16.5.11.1.	Methylomonas	0	1	0	0	1	0
6	0.2.16.5.11.1.	Methylosoma	0	1	0	0	1	0
6	0.2.16.5.11.1.	Methylosphaera	0	1	0	0	1	0
4	0.2.16.5.12	Oceanospirillales	5	19	0	1	18	0
5	0.2.16.5.12.1	Alcanivoracaceae	1	1	0	0	1	0
6	0.2.16.5.12.1.	Kangiella	0	1	0	0	1	0
5	0.2.16.5.12.2	Hahellaceae	2	2	0	0	2	0
6	0.2.16.5.12.2.	Endozoicomonas	0	1	0	0	1	0
6	0.2.16.5.12.2.	Zooshikella	0	1	0	0	1	0
5	0.2.16.5.12.3	Halomonadaceae	7	7	0	0	7	0
6	0.2.16.5.12.3.	Aidingimonas	0	1	0	0	1	0

6	0.2.16.5.12.3	Cobetia	0	1	0	0	1	0
6	0.2.16.5.12.3	Halomonas	0	1	0	0	1	0
6	0.2.16.5.12.3	Halotalea	0	1	0	0	1	0
6	0.2.16.5.12.3	Halovibrio	0	1	0	0	1	0
6	0.2.16.5.12.3	Modicisalibacter	0	1	0	0	1	0
6	0.2.16.5.12.3	Salinicola	0	1	0	0	1	0
5	0.2.16.5.12.4	Oceanospirillaceae	8	8	0	1	7	0
6	0.2.16.5.12.4	Balneatrix	0	1	0	0	1	0
6	0.2.16.5.12.4	Bermanella	0	1	0	0	1	0
6	0.2.16.5.12.4	Nitrincola	0	1	0	1	0	0
6	0.2.16.5.12.4	Oceaniserpentilla	0	1	0	0	1	0
6	0.2.16.5.12.4	Oceanobacter	0	1	0	0	1	0
6	0.2.16.5.12.4	Oleispira	0	1	0	0	1	0
6	0.2.16.5.12.4	Pseudospirillum	0	1	0	0	1	0
6	0.2.16.5.12.4	Reinekea	0	1	0	0	1	0
5	0.2.16.5.12.5	Oceanospirillales_incertae_sedis	1	1	0	0	1	0
6	0.2.16.5.12.5	Spongiispira	0	1	0	0	1	0
4	0.2.16.5.13	Pasteurellales	1	5	0	1	4	0
5	0.2.16.5.13.1	Pasteurellaceae	5	5	0	1	4	0
6	0.2.16.5.13.1	Bibersteinia	0	1	0	1	0	0
6	0.2.16.5.13.1	Chelonobacter	0	1	0	0	1	0
6	0.2.16.5.13.1	Haemophilus	0	1	0	0	1	0
6	0.2.16.5.13.1	Mannheimia	0	1	0	0	1	0
6	0.2.16.5.13.1	Phocoenobacter	0	1	0	0	1	0
4	0.2.16.5.14	Pseudomonadales	2	4	1	0	4	0
5	0.2.16.5.14.1	Moraxellaceae	2	2	0	0	2	0
6	0.2.16.5.14.1	Acinetobacter	0	1	0	0	1	0
6	0.2.16.5.14.1	Perlucidibaca	0	1	0	0	1	0
5	0.2.16.5.14.2	Pseudomonadaceae	2	2	1	0	2	0
6	0.2.16.5.14.2	Azorhizophilus	0	1	0	0	1	0
6	0.2.16.5.14.2	Pseudomonas	0	1	1	0	1	0
4	0.2.16.5.15	Thiotrichales	3	7	3	0	6	1
5	0.2.16.5.15.1	Piscirickettsiaceae	4	4	0	0	4	0
6	0.2.16.5.15.1	Cycloclasticus	0	1	0	0	1	0
6	0.2.16.5.15.1	Hydrogenovibrio	0	1	0	0	1	0
6	0.2.16.5.15.1	Methylophaga	0	1	0	0	1	0
6	0.2.16.5.15.1	Sulfurivirga	0	1	0	0	1	0
5	0.2.16.5.15.2	Thiotrichaceae	1	1	1	0	1	0
6	0.2.16.5.15.2	Beggiatoa	0	1	1	0	1	0
5	0.2.16.5.15.3	Thiotrichales_incertae_sedis	2	2	2	0	1	1
6	0.2.16.5.15.3	Caedibacter	0	1	1	0	0	0
6	0.2.16.5.15.3	Fangia	0	1	1	0	1	1
4	0.2.16.5.16	Xanthomonadales	2	15	1	0	14	3
5	0.2.16.5.16.1	Sinobacteraceae	5	5	1	0	5	0
6	0.2.16.5.16.1	Alkanibacter	0	1	0	0	1	0
6	0.2.16.5.16.1	Hydrocarboniphaga	0	1	0	0	1	0
6	0.2.16.5.16.1	Nevskia	0	1	0	0	1	0
6	0.2.16.5.16.1	Singularimonas	0	1	1	0	1	0
6	0.2.16.5.16.1	Steroidobacter	0	1	0	0	1	0
5	0.2.16.5.16.2	Xanthomonadaceae	10	10	0	0	9	3
6	0.2.16.5.16.2	Aquimonas	0	1	0	0	1	1
6	0.2.16.5.16.2	Dokdonella	0	1	0	0	1	0
6	0.2.16.5.16.2	Dyella	0	1	0	0	1	0
6	0.2.16.5.16.2	Frateuria	0	1	0	0	0	1
6	0.2.16.5.16.2	Fulvimonas	0	1	0	0	1	1
6	0.2.16.5.16.2	Luteimonas	0	1	0	0	1	0
6	0.2.16.5.16.2	Pseudoxanthomonas	0	1	0	0	1	0
6	0.2.16.5.16.2	Rhodanobacter	0	1	0	0	1	0
6	0.2.16.5.16.2	Rudaea	0	1	0	0	1	0
6	0.2.16.5.16.2	Stenotrophomonas	0	1	0	0	1	0
2	0.2.17	Spirochaetes	1	2	0	0	2	0
3	0.2.17.1	Spirochaetes	1	2	0	0	2	0
4	0.2.17.1.1	Spirochaetales	1	2	0	0	2	0
5	0.2.17.1.1.1	Leptospiraceae	2	2	0	0	2	0
6	0.2.17.1.1.1.1	Leptonema	0	1	0	0	1	0
6	0.2.17.1.1.1.2	Turneriella	0	1	0	0	1	0
2	0.2.18	Synergistetes	1	6	0	1	4	4
3	0.2.18.1	Synergistia	1	6	0	1	4	4
4	0.2.18.1.1	Synergistales	1	6	0	1	4	4
5	0.2.18.1.1.1	Synergistaceae	6	6	0	1	4	4
6	0.2.18.1.1.1.1	Aminiphilus	0	1	0	1	0	1
6	0.2.18.1.1.1.2	Aminomonas	0	1	0	0	1	1
6	0.2.18.1.1.1.3	Jonquetella	0	1	0	0	1	0
6	0.2.18.1.1.1.4	Pyramidobacter	0	1	0	0	1	1
6	0.2.18.1.1.1.5	Synergistes	0	1	0	0	0	1
6	0.2.18.1.1.1.6	Thermovirga	0	1	0	0	1	0
2	0.2.19	Tenericutes	1	1	0	1	1	1
3	0.2.19.1	Mollicutes	1	1	0	1	1	1
4	0.2.19.1.1	Anaeroplasmatales	1	1	0	1	1	1
5	0.2.19.1.1.1	Anaeroplasmataceae	1	1	0	1	1	1
6	0.2.19.1.1.1.1	Asteroleplasma	0	1	0	1	1	1
2	0.2.20	Thermodesulfobacteria	1	2	0	2	2	0
3	0.2.20.1	Thermodesulfobacteria	1	2	0	2	2	0
4	0.2.20.1.1	Thermodesulfobacteriales	1	2	0	2	2	0
5	0.2.20.1.1.1	Thermodesulfobacteriaceae	2	2	0	2	2	0
6	0.2.20.1.1.1.1	Caldimicrobium	0	1	0	1	1	0
6	0.2.20.1.1.1.2	Thermodesulfatator	0	1	0	1	1	0

2	0.2.21	Thermotogae	1	2	0	0	0	2
3	0.2.21.1	Thermotogae	1	2	0	0	0	2
4	0.2.21.1.1	Thermotogales	1	2	0	0	0	2
5	0.2.21.1.1.1	Thermotogaceae	2	2	0	0	0	2
6	0.2.21.1.1.1.1	Geotoga	0	1	0	0	0	1
6	0.2.21.1.1.1.2	Kosmotoga	0	1	0	0	0	1
2	0.2.22	Verrucomicrobia	4	10	5	1	4	2
3	0.2.22.1	Opitutae	2	3	0	0	2	2
4	0.2.22.1.1	Opitutales	1	2	0	0	2	1
5	0.2.22.1.1.1	Opitutaceae	2	2	0	0	2	1
6	0.2.22.1.1.1.1	Alterococcus	0	1	0	0	1	0
6	0.2.22.1.1.1.2	Opitutus	0	1	0	0	1	1
4	0.2.22.1.2	Puniceicoccales	1	1	0	0	0	1
5	0.2.22.1.2.1	Puniceicoccaceae	1	1	0	0	0	1
6	0.2.22.1.2.1.1	Cerasicoccus	0	1	0	0	0	1
3	0.2.22.2	Spartobacteria	1	1	1	0	0	0
4	0.2.22.2.1	unclassified	1	1	1	0	0	0
5	0.2.22.2.1.1	unclassified	1	1	1	0	0	0
6	0.2.22.2.1.1.1	unclassified	0	1	1	0	0	0
3	0.2.22.3	Subdivision3	1	1	1	0	0	0
4	0.2.22.3.1	unclassified	1	1	1	0	0	0
5	0.2.22.3.1.1	unclassified	1	1	1	0	0	0
6	0.2.22.3.1.1.1	unclassified	0	1	1	0	0	0
3	0.2.22.4	Verrucomicrobiae	1	5	3	1	2	0
4	0.2.22.4.1	Verrucomicrobiales	1	5	3	1	2	0
5	0.2.22.4.1.1	Verrucomicrobiaceae	5	5	3	1	2	0
6	0.2.22.4.1.1.1	Akkermansia	0	1	0	0	1	0
6	0.2.22.4.1.1.2	Luteolibacter	0	1	0	1	0	0
6	0.2.22.4.1.1.3	Persicirhabdus	0	1	1	0	1	0
6	0.2.22.4.1.1.4	Prosthecobacter	0	1	1	0	0	0
6	0.2.22.4.1.1.5	Verrucomicrobium	0	1	1	0	0	0
2	0.2.23	Bacteria_incertae_sedis	1	1	0	0	1	0
3	0.2.23.1	Ktedonobacteria	1	1	0	0	1	0
4	0.2.23.1.1	Ktedonobacterales	1	1	0	0	1	0
5	0.2.23.1.1.1	Ktedonobacteraceae	1	1	0	0	1	0
6	0.2.23.1.1.1.1	Ktedonobacter	0	1	0	0	1	0
2	0.2.24	Cyanobacteria	1	1	0	0	0	1
3	0.2.24.1	Cyanobacteria	1	1	0	0	0	1
4	0.2.24.1.1	Cyanobacteria_order_incertae_	1	1	0	0	0	1
5	0.2.24.1.1.1	Chloroplast	1	1	0	0	0	1
6	0.2.24.1.1.1.1	Chlorarachniophyceae	0	1	0	0	0	1
2	0.2.25	OP10	1	1	1	0	1	0
3	0.2.25.1	unclassified	1	1	1	0	1	0
4	0.2.25.1.1	unclassified	1	1	1	0	1	0
5	0.2.25.1.1.1	unclassified	1	1	1	0	1	0
6	0.2.25.1.1.1.1	unclassified	0	1	1	0	1	0
